# Using in vivo oxidation status of one- and two-component redox relays to determine H_2_O_2_ levels linked to signaling and toxicity

**DOI:** 10.1186/s12915-018-0523-6

**Published:** 2018-06-01

**Authors:** Alba Domènech, José Ayté, Fernando Antunes, Elena Hidalgo

**Affiliations:** 1 Department of Experimental and Health Sciences, Universitat Pompeu Fabra, C/ Dr. Aiguader 88, 08003 Barcelona, Spain; 2 Departamento de Química e Bioquímica and Centro de Química e Bioquímica, Faculdade de Ciências, Universidade de Lisboa, Lisbon, Portugal

**Keywords:** Thiol switch, H_2_O_2_ sensor, Peroxiredoxin, OxyR, Pap1, H_2_O_2_ gradients

## Abstract

**Background:**

Hydrogen peroxide (H_2_O_2_) is generated as a by-product of metabolic reactions during oxygen use by aerobic organisms, and can be toxic or participate in signaling processes. Cells, therefore, need to be able to sense and respond to H_2_O_2_ in an appropriate manner. This is often accomplished through thiol switches: Cysteine residues in proteins that can act as sensors, and which are both scarce and finely tuned. Bacteria and eukaryotes use different types of such sensors—either a one-component (OxyR) or two-component (Pap1-Tpx1) redox relay, respectively. However, the biological significance of these two different signaling modes is not fully understood, and the concentrations and peroxides driving those types of redox cascades have not been determined, nor the intracellular H_2_O_2_ levels linked to toxicity. Here we elucidate the characteristics, rates, and dynamic ranges of both systems.

**Results:**

By comparing the activation of both systems in fission yeast, and applying mathematical equations to the experimental data, we estimate the toxic threshold of intracellular H_2_O_2_ able to halt aerobic growth, and the temporal gradients of extracellular to intracellular peroxides. By calculating both the oxidation rates of OxyR and Tpx1 by peroxides, and their reduction rates by the cellular redoxin systems, we propose that, while Tpx1 is a sensor and an efficient H_2_O_2_ scavenger because it displays fast oxidation and reduction rates, OxyR is strictly a H_2_O_2_ sensor, since its reduction kinetics are significantly slower than its oxidation by peroxides, and therefore, it remains oxidized long enough to execute its transcriptional role. We also show that these two paradigmatic H_2_O_2_-sensing models are biologically similar at pre-toxic peroxide levels, but display strikingly different activation behaviors at toxic doses.

**Conclusions:**

Both Tpx1 and OxyR contain thiol switches, with very high reactivity towards peroxides. Nevertheless, the fast reduction of Tpx1 defines it as a scavenger, and this efficient recycling dramatically changes the Tpx1-Pap1 response to H_2_O_2_ and connects H_2_O_2_ sensing to the redox state of the cell. In contrast, OxyR is a true H_2_O_2_ sensor but not a scavenger, being partially insulated from the cellular electron donor capacity.

**Electronic supplementary material:**

The online version of this article (10.1186/s12915-018-0523-6) contains supplementary material, which is available to authorized users.

## Background

The use of oxygen by aerobic organisms in several metabolic reactions has the side effect of the toxicity associated with oxygen by-products such as hydrogen peroxide (H_2_O_2_). The possible toxic effects exerted by oxygen-derived species are counteracted with antioxidant systems, so that a proper intracellular environment is maintained. In addition to this toxicity, decades of work support the participation of H_2_O_2_ in signaling events, based on its capacity to diffuse in space and through membranes, and its discriminate reactivity towards specific cysteine (Cys) residues in proteins.

The toxicity associated with H_2_O_2_ may be due to direct inactivation of enzymes or to its conversion to the more reactive species hydroxyl radical, and it normally leads to irreversible modifications of protein backbones or of amino acid side chains. However, the direct oxidation of Cys and, to a lesser extent, methionine residues with H_2_O_2_ can give rise to reversible modifications, such as sulfenic acid or disulfides or as methionine sulfoxides, respectively, which can be reduced by cellular activities. Reversible Cys oxidation has been unambiguously shown to participate in the activation of H_2_O_2_ signaling cascades. However, free Cys, small thiols, and most Cys in proteins react quite poorly with H_2_O_2_, with rate constants of the order of 10 M^− 1^ s^− 1^ [1], indicating that their oxidation cannot be accomplished during rapid physiological peroxide fluctuations. Thus, Cys in proteins must display low pKa, to enhance the thiolate fraction, as a prerequisite for fast and efficient oxidation by peroxides, although its nucleophilicity (to attack the H_2_O_2_ electrophile) and its capacity to stabilize both the transition state with the reactant, H_2_O_2_, and the leaving group (which occurs after the rupture of the peroxidic bond) also has to be preserved [2–4].

Few Cys in proteins fulfill those premises, and have as a consequence exquisite sensitivity and specificity to react with H_2_O_2_. The fast and reversible reaction of peroxides with those Cys residues in proteins, two conditions for signaling, provides the basis by which H_2_O_2_ triggers intracellular signaling cascades [5–7]. These Cys residues in specific proteins are called thiol switches, since they can switch the activity of the H_2_O_2_ sensor protein on and off. The high reactivity of these thiol switches with peroxides (with rate constants ranging from 10^5^ to 10^7^ M^− 1^ s^− 1^ [8]), along with the ability of H_2_O_2_ to diffuse through membranes, make H_2_O_2_ one of the oxidants better suited for signaling. Even though some bioinformatic tools have been proposed to predict Cys reactivity in silico [9], currently real H_2_O_2_ protein sensors can be demonstrated only experimentally, and this has been accomplished with the pioneer work on bacterial OxyR [10], and the later characterization of the eukaryotic redox relays Gpx3-Yap1 [11] and Tpx1-Pap1 [12, 13].

**Fig. 1:**
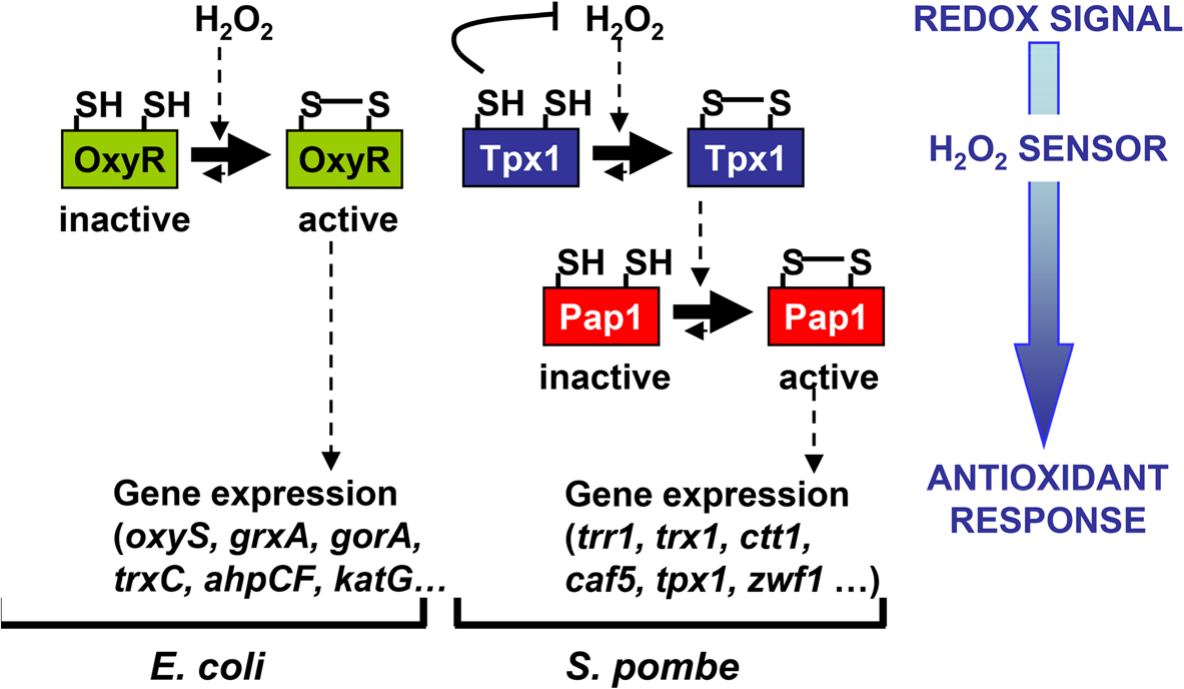
Scheme depicting the two types of H_2_O_2_-sensing modules: the one-component OxyR and the two-component Tpx1-Pap1 systems. Some of the genes regulated by these systems are indicated. See text for details. *E. coli Escherichia coli*, *S. pombe Schizosaccharomyces pombe*

The transcription factor OxyR, which has a reaction rate with H_2_O_2_ of 1.1–1.7 × 10^5^ M^− 1^ s^− 1^ [14, 15], is a tetramer that binds to the promoter of target antioxidant genes before and after stress sensing [16]. Upon activation by peroxides, OxyR suffers conformational changes, becoming a potent activator of transcription [17]. Low concentrations of H_2_O_2_ are sufficient to activate OxyR fully and transiently through Cys oxidation to a disulfide bond [10, 14] (Fig. 1). Glutaredoxin (Grx) 1, with the glutathione (GSH)-GSH reductase-NADPH as the electron donor system, reduces the transcription factor back to the inactive conformation [10]. Although some other models have been proposed for oxidation by other agents [18–20], it is widely accepted that H_2_O_2_ reacts with the thiol switch Cys199 in OxyR to yield an unstable sulfenic acid, which reacts with Cys208 to form a metastable intramolecular disulfide bond [10]. In fact, the fast, reversible, and specific interaction of OxyR with H_2_O_2_ has been exploited in the design of HyPer, a genetically encoded biosensor that enables real-time imaging of peroxides in living cells, and which is based on the insertion of a yellow fluorescent protein into the sequence of OxyR [21, 22].

In eukaryotes, the activation of signaling cascades uses a different strategy. The redox-dependent transcription factors Yap1 in *Saccharomyces cerevisiae* and Pap1 in *Schizosaccharomyces pombe* rely on upstream components, the real H_2_O_2_ sensors, to sense oxidative stress: the glutathione peroxidase Gpx3 and the peroxiredoxin (Prx) Tpx1, respectively [11–13] (Fig. 1). In *S. pombe*, Tpx1 has also been described as the main peroxide scavenger during aerobic growth, so that cells lacking this Prx can grow only under anaerobic conditions on solid plates [23]. Another interesting aspect of Tpx1 and most eukaryotic Prxs is their inactivation by high H_2_O_2_ levels through hyper-oxidation of the reactive Cys to sulfinic acid (SO_2_H), allowing a temporal build-up of peroxides and a lack of activation of Pap1 [12, 13]. Importantly, Prxs are inactivated while engaged in their catalytic cycles, with higher inactivation values as the reaction rates increase [24]. As with most Prxs, the intracellular concentration of the Tpx1 dimer is very high, around 3.5 μM (2 × 10^5^ copies per cell) according to proteome-wide studies [25]. An interesting proposal by Winterbourn and Hampton is that the highly abundant Prxs, upon oxidation by peroxides, may trigger the otherwise weak oxidation not only of signaling cascades but also of the general thiol proteome [8]. Nevertheless, direct oxidation of proteins harboring low-reactive thiols may proceed supported by localized H_2_O_2_ pools and specific protein interactions [26].

To describe H_2_O_2_ sensing and signaling unambiguously by the one- and two-component models described so far, we have expressed bacterial OxyR in fission yeast at a concentration similar to that of Tpx1. We first confirmed that OxyR oxidation does not require Prxs, and that in *S. pombe* it depends on the thioredoxin (Trx) system for recycling, although the kinetics of oxidation by peroxides are significantly faster than its reduction, and concentration of peroxides is prevalent in the presence of reduced Trxs. At higher doses of peroxides, the hyper-oxidation of Tpx1 unlinks Pap1 activation from peroxide concentrations, inducing a dual temporal wave of Pap1 oxidation; the first wave of activation is too transient to trigger an antioxidant response. Since in our system OxyR strictly responds to intracellular peroxides without affecting their concentration, we determined the intracellular steady-state concentrations of H_2_O_2_ based on OxyR oxidation in cells lacking Tpx1, and concluded that 0.3 μM of intracellular peroxides can halt the growth of *S. pombe* on solid plates. By applying mathematical equations to our experimental data, we demonstrate that the permeability of H_2_O_2_ allows an extracellular-to-intracellular peroxide gradient of around 40-fold at sub-toxic levels (100–200 μM), but peroxide scavenging, mainly driven by Tpx1 at low doses of peroxides and initial time points, generates transient gradients of 300-fold. We fully describe the common and specific features of these two types of redox relays, and use their oxidation kinetics to define the H_2_O_2_ intracellular gradients, the cellular scavenging activities governing at different peroxide ranks, the redox state of the Trx-reducing system, and the status of both signaling cascades. We also discuss how these two types of redox relays impact the modularity of H_2_O_2_ sensing and its insulation from the redox state of the cell.

## Results

### Expression and oxidation of bacterial OxyR in eukaryotic cells

**Fig. 2:**
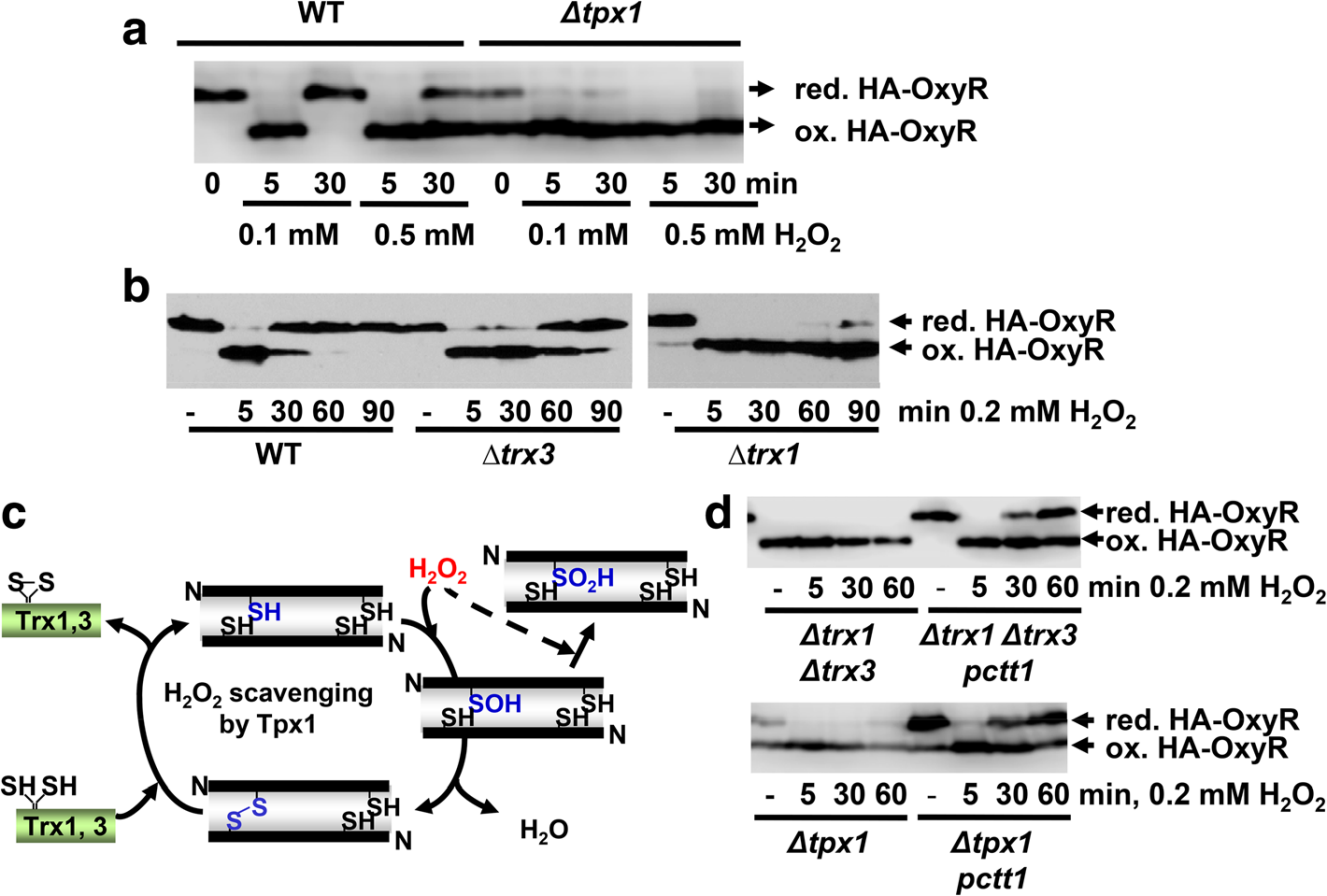
Expression of *E. coli’*s OxyR in yeast cells. **a** Tpx1-independent oxidation of OxyR in fission yeast. MM cultures of strains AD29 (WT) and AD36 (*Δtpx1*), carrying an integrative *sty1* promoter-driven *HA-oxyR* gene, were treated or not with 0.1 mM or 0.5 mM H_2_O_2_ for the times indicated. TCA/AMS protein extracts were obtained, processed by SDS-PAGE, and analyzed by Western blot using antibodies against HA. Reduced (red.) and oxidized (ox.) HA-OxyR are indicated with arrows. **b** The Trx system participates in OxyR reduction. MM cultures of strains AD29 (WT), AD47 (*Δtrx3*), and AD61 (*Δtrx1*), all constitutively expressing HA-OxyR, were treated or not with 0.2 mM H_2_O_2_ for the times indicated. TCA/AMS extracts were prepared and analyzed as described in (**b**) **c** H_2_O_2_ scavenging by Tpx1, and the role of Trx1 and Trx3 in the recycling of disulfide-link Tpx1. **d** Cells lacking Tpx1, or both Trx1 and Trx3, display constitutive levels of oxidized OxyR. Overexpression of Ctt1 suppresses basal OxyR oxidation in cells lacking Tpx1 or cytosolic Trxs. TCA extracts of MM cultures of the HA-OxyR-expressing strains AD62 (*Δtrx1 Δtrx3*), AD98 + p464 (*Δtrx1 Δtrx3 pctt1*; it overexpresses catalase from plasmid p464) and AD36 (*Δtpx1*), AD94 + p464 (*Δtpx1 pctt1*; it overexpresses catalase from plasmid p464) treated or not with 0.2 mM H_2_O_2_ for the times indicated, were processed and analyzed as in (**b**) AMS 4-acetamido-4′-maleimidylstilbene-2,2′-disulfonic acid, *E. coli Escherichia coli*, MM minimal medium, ox. oxidized, red. reduced, SDS-PAGE sodium dodecyl sulphate-polyacrylamide gel electrophoresis, TCA trichloroacetic acid, WT wild type

Bacterial OxyR oxidation by peroxides was described 15 years earlier than the peroxidase-dependent activation of eukaryotic transcription factors (Fig. 1). To confirm the properties and kinetics of OxyR, we expressed HA-tagged OxyR in fission yeast at levels similar to those of the H_2_O_2_ sensor Tpx1. As shown in Fig. 2a, full and transient OxyR oxidation can be accomplished at 0.1 mM extracellular peroxides. Tpx1 is not required for OxyR oxidation (Fig. 2a, *Δtpx1* + HA-OxyR), and a strain lacking all four *S. pombe* thioredoxin peroxidases (Tpx1, Gpx1, Pmp20, and Bcp1) is still capable of oxidizing OxyR (Additional file 1: Figure S1a), confirming that OxyR contains a true thiol switch. In fact, deletion of Tpx1 promotes OxyR oxidation either in the presence of H_2_O_2_ or under basal conditions (Fig. 2a; see below). Cells possess two major cascades, the Trx and the Grx/GSH systems, to maintain thiols in their reduced form, and to reduce disulfides formed upon oxidant exposure or after specific enzymatic reactions [27–31]. The *S. pombe* genome contains genes coding for two cytosolic Trxs, one dithiol cytosolic Grx, one Trx reductase (Trr1), and one GSSG reductase (Pgr1) [32]. We performed long kinetics after H_2_O_2_ imposition, and observed that OxyR returned to the reduced form after 30–60 min of 200 μM stress (Fig. 2b), when significant levels of H_2_O_2_ are still present (see below, in Section 2.3), indicating that heterologous OxyR is being actively reduced by the *S. pombe* reducing systems. While the absence of Grx1 or Pgr1 had no effect on the basal levels of OxyR, or on its reduction after H_2_O_2_ stress (Additional file 1: Figure S1b), the individual deletion of either of the genes coding for cytosolic Trxs, *trx1* or *trx3*, had a long-term impact on the recycling of OxyR after stress imposition (Fig. 2b). In particular, OxyR remained oxidized 90 min after H_2_O_2_ stress in *∆trx1* cells. The absence of both cytosolic thioredoxins Trx1 and Trx3 triggers constitutive oxidation of OxyR (Fig. 2d, top panel). Therefore, two strain backgrounds (*∆tpx1* and *∆trx1 ∆trx3*) display constitutively significant oxidized OxyR in the absence of added peroxides. However, these results are not direct evidence for the role of the Trx branch in the reduction of OxyR. As mentioned in the Section 1, the Prx Tpx1 is the main scavenger of H_2_O_2_ during aerobic growth [23], and cytosolic Trx1 and Trx3 recycle disulfide-linked Tpx1 dimers at the expense of reduced cofactor [33] (Fig. 2c). The Tpx1 cycle can be poisoned at high concentrations of peroxides through hyper-oxidation of the sulfenic acid form (SOH) to sulfinic acid (SO_2_H) [12, 13] (Fig. 2c). We hypothesized that in both strains, *∆tpx1* and *∆trx1 ∆trx3*, H_2_O_2_ scavenging may be compromised, and that enhanced basal peroxides could trigger OxyR oxidation. Indeed, ectopic overexpression of catalase in *∆tpx1* or *∆trx1 ∆trx3* strains fully suppresses OxyR oxidation under basal conditions (Fig. 2d), indicating that OxyR is a sensitive and specific sensor of H_2_O_2_ levels.

### Using Tpx1 and OxyR oxidation kinetics to extrapolate intracellular concentrations of H_2_O_2_: an initial 40:1 gradient across membranes is transiently enhanced to 250:1 due to Tpx1 scavenging

The oxidation/reduction kinetics of protein sensors in signaling cascades can be used to calculate extracellular-to-intracellular H_2_O_2_ gradients by combining biological data and applied equations. Our biological system, with one cell type expressing the two models of H_2_O_2_-sensing redox relays, and our experimental methodology, which facilitates the in vivo trapping of the thiol redox states within second frames, constitutes a superb model to apply kinetic equations to extrapolate intracellular H_2_O_2_ levels.

**Fig. 3:**
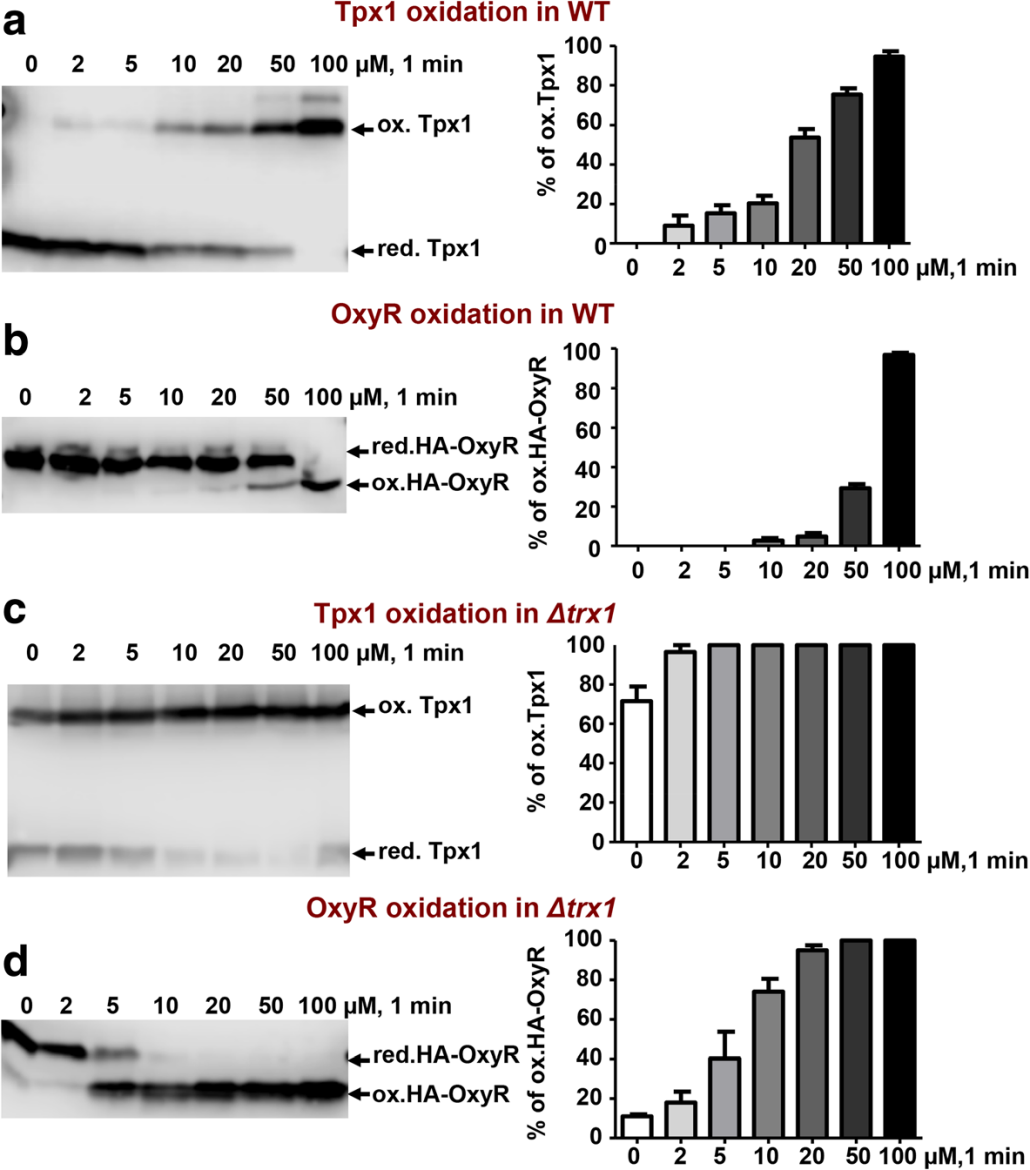
Kinetics of Tpx1 and HA-OxyR oxidation in wild-type and *Δtrx1* cells*.* Cultures of strains AD29 (WT) (**a**, **b**) or AD61 (*Δtrx1*) (**c**, **d**), expressing HA-OxyR, were treated or not with the indicated concentrations of H_2_O_2_ for 1 min. Protein extracts were obtained and processed as described in Fig. 1b using antibodies against Tpx1 (**a, c**) or HA (**b**, **d**). Reduced and oxidized Tpx1 and OxyR are indicated with arrows. The percentages of oxidized Tpx1 or HA-OxyR are indicated with bars (right panels). Error bars (standard error of the mean) from three independent biological replicates are shown. WT wild type

We experimentally applied different concentrations of H_2_O_2_ to cultures of a wild-type background expressing HA-OxyR and obtained protein extracts at different time points. We measured by Western blot the percentage of Tpx1 and OxyR oxidation after 1 min of applying increasing extracellular concentrations of H_2_O_2_ (Fig. 3a, b), or changing times at fixed peroxide levels (Additional file 2: Figure S2). As shown in Fig. 3a, oxidized covalent Tpx1 dimer can be observed at 2 μM extracellular H_2_O_2_, and it only reaches full oxidation at 100 μM peroxides. This pattern of Tpx1 oxidation is identical to that of wild-type cells not expressing HA-OxyR (data not shown). OxyR oxidation does not occur till we apply a dose of 20 μM, which can be explained by the lower second-order rates of OxyR oxidation relative to those of Prxs, but full OxyR oxidation is reached also at 100 μM (Fig. 3b). We then measured OxyR oxidation in the absence of Trx1, which jeopardizes H_2_O_2_ scavenging by Tpx1 since the Prx cannot be recycled (Fig. 2c). In *Δtrx1* cells, OxyR oxidation occurs at very low concentrations of extracellular H_2_O_2_ (Fig. 3d), probably due to the accumulation of intracellular peroxides in this strain background since the main H_2_O_2_ scavenger Tpx1 is not being recycled (Fig. 2c). Very similar OxyR oxidation kinetics can be observed in *Δtpx1* cells under anaerobic conditions (Additional file 2: Figure S2a).

Applying a second-order rate constant of 1.4 × 10^5^ M^− 1^ s^− 1^ for OxyR [14, 15], a gradient of 267 ± 8 (*n* = 2) is obtained by fitting Eq. 1 (see Methods) to the profile of OxyR oxidation observed with H_2_O_2_ extracellular concentrations of 20 and 50 μM in wild-type cells (Additional file 2: Figure S2a and Additional file 3: Figure S3a). For lower H_2_O_2_ concentrations, this gradient should be higher, as at H_2_O_2_ extracellular concentrations of 20 and 50 μM some Tpx1 oxidation is already observed (Fig. 3 and Additional file 2: Figure S2b). Unfortunately, it was not possible to determine the gradient for lower H_2_O_2_ extracellular concentrations, because OxyR oxidation was not detected.

When Tpx1 is deleted (the *Δtpx1* strain), a gradient of 40 ± 9 (*n* = 6) is obtained under anaerobic conditions, based on profiles of OxyR oxidation observed with extracellular H_2_O_2_ in the 2–100 μM range (Additional file 2: Figure S2a and Additional file 3: Figure S3b). This suggests the important role of Tpx1 as a driving force for large H_2_O_2_ gradients, which is further supported by the gradient of 42 ± 12 (*n* = 5) observed in the strain *Δtrx1* under aerobic conditions for H_2_O_2_ extracellular concentrations between 2 and 50 μM (Additional file 2: Figure S2a and Additional file 3: Figure S3a), in which Tpx1 recycling is compromised [33]. The gradients obtained in *Δtpx1* and *Δtrx1* strains are likely due to catalase activity (see below).

### Using extracellular concentrations of H_2_O_2_ to corroborate peroxide gradients across membranes and the role of Tpx1 and catalase at different concentrations of applied peroxides

The gradients measured in the previous section were based on the oxidation profile of OxyR observed after addition of H_2_O_2_. In this section, we independently corroborate these determinations by measuring gradients based on the extracellular consumption of H_2_O_2_. We measured extracellular H_2_O_2_ levels in cultures of wild-type, *Δctt1*, *Δtpx1*, or *Δtpx1 Δctt1* strains after addition of different concentration of peroxides. As shown in Additional file 4: Figure S4, the main intracellular scavenger of peroxides after addition of a toxic concentration of H_2_O_2_, 0.5 mM, is catalase. However, at sub-toxic doses of peroxides such as 0.1 mM, both Tpx1 and Ctt1 seem to have an overlapping capacity to scavenge peroxides, since single-deletion mutants *Δctt1* or *Δtpx1* have a detoxifying capacity similar to that of a wild-type strain, while the decay of extracellular peroxides of *Δtpx1 Δctt1* cultures is identical to that of a cell-free system (Additional file 4: Figure S4).

Gradients estimated from the ratio between the rate of removal of intracellular H_2_O_2_ and the rate of removal of extracellular H_2_O_2_ are shown in Table 1. At 0.5 mM H_2_O_2_, the pseudo-first-order rate constant (*k*_cons_) that characterizes the kinetics of H_2_O_2_ consumption was 0.16 s^− 1^. As referred above, under these conditions, catalase is the only antioxidant responsible for the removal of H_2_O_2_, and so it was assumed that the intracellular removal of H_2_O_2_ is 6 s^− 1^ (Table 2). Thus, a catalase-driven gradient of 38 for the wild-type strain (Table 1) when cells are subjected to 0.5 mM was obtained. For the *Δtpx1* strain, catalase is probably the main intracellular sink for H_2_O_2_ when cells are exposed to either 0.1 or 0.5 mM and generates gradients of 27 and 33, respectively (Table 1). These catalase-driven gradients are like those determined from OxyR oxidation profiles in *Δtpx1* and *Δtrx1* (Table 3), suggesting that in these strains, catalase is the main sink of H_2_O_2_. A reliable determination of gradients driven by Tpx1 was not possible from H_2_O_2_ consumption profiles, since at 100 μM, Tpx1 already shows very high levels of oxidation (Fig. 3 and Additional file 2: Figure S2b). This may also explain the apparent overlapping capacity of Tpx1 and Ctt1 to consume H_2_O_2_ shown in Additional file 4: Figure S4.

**Table 1:** Determination of pseudo-first-order rate constants for the extracellular removal of H_2_O_2_ and determination of catalase-driven gradients

Extracellular removal of H_2_O_2_ (*k*_cons_) (s^− 1^)			
	H_2_O_2_ = 0.5 mM	H_2_O_2_ = 0.1 mM	
Wild type	0.16	0.27	
*Δtpx1*	0.18	0.22	
*Δctt1*	0	0.24	
Catalase-driven gradient			
	*k*_cons_ (s^−1^)	*k*_intracellular_ (s^− 1^)	Gradient
Wild type	0.16	6.0	38
*Δtpx1* (H_2_O_2_ = 0.5 mM)	0.18	6.0	33
*Δtpx1* (H_2_O_2_ = 0.1 mM)	0.22	6.0	27

**Table 2:** Calculation of the overall pseudo-first-order rate constant for intracellular consumption of H_2_O_2_

	[Protein] (μM)^a^	Second-order rate constant (M^− 1^ s^− 1^)	Pseudo-first-order rate constant (s^− 1^)
Catalase	0.13	4.6 × 10^7 b^	*k*_cat_ = 6.0
Tpx1	3.5	1.0 × 10^7 c^	*k*_*tpx1*_ = 35
Overall			*k*_catabolism_ = 41

^a^Values extracted from [25]

^b^Values extracted from [45]

^c^Values extracted from [8]

**Table 3:** Comparison of gradients obtained from OxyR oxidation and H_2_O_2_ consumption experiments

	OxyR oxidation			H_2_O_2_ consumption		
Strain	Wild type (*n* = 2)	*Δtpx1* (*n* = 6)	*Δtrx1* (*n* = 5)	Wild type (*n* = 3)	*Δtpx1* (*n* = 3)	*Δtpx1* (*n* = 3)
H_2_O_2_ (μM)	20–50	2–100	2–50	500	500	100
Gradient	267 ± 8	40 ± 9	42 ± 12	38	33	27

### Disentangling OxyR reduction from oxidation and calculation of intracellular toxic levels of H_2_O_2_

Here we analyze how the heterologous expressed OxyR is a functional partner of the redox networks in *S. pombe*. In Section 2.1, it was not clear whether the Trx1/Trx3 branch was able to reduce OxyR, because the increased oxidation of OxyR observed in mutants for these pathways could not be directly ascribed to a deficient OxyR reduction. For example, in the *Δtrx1* strain, Tpx1 activity is compromised, leading to increased levels of H_2_O_2_. Thus, the increased OxyR oxidation observed in this strain could be caused either by its increased oxidation or by its impaired reduction. Based on the OxyR oxidation profiles and the gradients determined before, we now address quantitatively the roles played by Trx1 and Trx3 in the reduction of OxyR. Oxidized OxyR is in a near steady state at 5 min (see Additional file 3: Figure S3), and so the rate of OxyR oxidation by H_2_O_2_ matches the rate of OxyR reduction by Trx system (Eqs. 2a and 2b in Methods). As can be seen in Table 4, to achieve similar levels of OxyR oxidation—24% and 28%, respectively, for the *Δtrx3* and *Δtrx1* strains—the levels of intracellular H_2_O_2_ need to be much higher in the *Δtrx3* strain (0.12 μM) than in the *Δtrx1* strain (0.048 μM).

**Table 4:** Analysis of reduction rates of heterologous expressed OxyR in *Schizosaccharomyces pombe*

	[H_2_O_2_] (μM)			Oxidation at steady state (5 min)^**b**^		*k*_switch-off_ (s^− **1**^)^**c**^	
Strain	Extracellular	Intracellular^**a**^	Gradient	OxyR	Tpx1	OxyR	Tpx1
*Δtrx1*	2	0.048	42	28%		0.017	
*Δtrx1*	5	0.12	42	89%		0.002	
*Δtrx3*	20	0.12	160	24%		0.056	
*Δtrx3*	50	0.31	160	77%		0.013	
Wild type	20	0.075	267	17%	46%	0.050	0.85
Wild type	50	0.19	267	58%	71%	0.019	0.79

^a^Intracellular concentration of H_2_O_2_ is calculated from the gradients between extracellular and intracellular H_2_O_2_ concentration determined from OxyR oxidation profiles

^b^The oxidation values for OxyR are extracted from experiments like those in Additional file 2: Figure S2 and the fitting shown in Additional file 3: Figure S3a, while Tpx1 oxidation values are an average of several biological replicates of experiments, like those of Additional file 2: Figure S2b

^c^The pseudo-first-order rate constant characterizing the reduction of thiol proteins is determined from the steady-state Eqs. 2 and 3, assuming that the second-order rate constants for the reaction of H_2_O_2_ with Tpx1 and OxyR are 1 × 10^7^ M^− 1^ s^− 1^ and 1.4 × 10^5^ M^− 1^ s^− 1^, respectively

Thus, for a much higher rate of OxyR oxidation in the *Δtrx3* strain, a similar level of OxyR oxidation is observed, indicating that OxyR is reduced much more efficiently in the *Δtrx3* strain. Likewise, for a similar H_2_O_2_ intracellular concentration of 0.12 μM in the *Δtrx3* and *Δtrx1* strains, the oxidation of OxyR is much higher in the *Δtrx1* strain (89%) than in the *Δtrx3* strain (24%). Therefore, for the same rate of oxidation, OxyR is reduced much less efficiently in the *Δtrx1* strain than in the *Δtrx3* strain. The predominant role of Trx1 for OxyR reduction is also seen from Additional file 5: Figure S5, in which the pattern of the relation between intracellular H_2_O_2_ and OxyR oxidation is similar in the wild-type and *Δtrx3* strains, while that of *Δtrx1* departs from wild-type behavior. From the highest values of *k*_switch-off_ determined in the *Δtrx1* and *Δtrx3* strains, it can be estimated that Trx1 contributes about 3.2 times more than Trx3 in the reduction of OxyR.

The reduction rates of Tpx1 and OxyR in the same strain can be compared with an equivalent rationale, based on the Tpx1 steady-state Eqs. 3a and 3b (see Methods). The maximal *k*_switch-off_ measured for Tpx1 is more than 20 times higher than that for OxyR (Table 4), indicating that reducing systems act more rapidly in Tpx1 than in OxyR. Such a higher efficiency probably reflects the true higher reactivity of Tpx1 with reducing systems rather than an inefficient reduction of OxyR by its non-native redox partners of *S. pombe*, because OxyR is also slowly reduced in bacteria [14]. In this regard, Prxs are characterized by a very high reactivity towards Trxs that are much higher when compared with other thiol proteins [26].

**Fig. 4:**
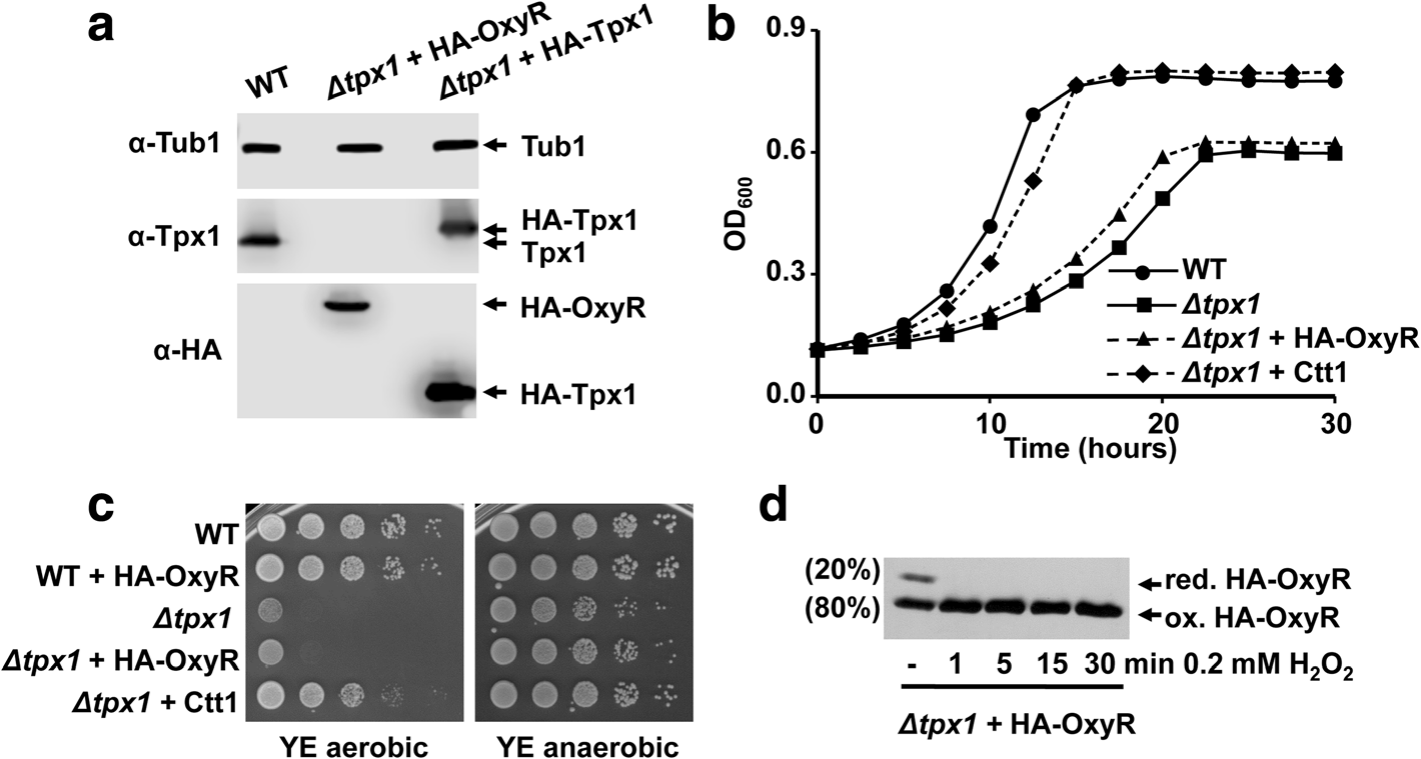
OxyR acts as a H_2_O_2_ sensor but not as a H_2_O_2_ scavenger. **a** Quantification of the intracellular concentration of OxyR expressed in fission yeast. TCA/AMS protein extracts were obtained from untreated MM cultures of strains 972 (WT), AD36 (*Δtpx1*) carrying an integrative *sty1* promoter-driven *HA-oxyR* gene, and SG5 (*Δtpx1*) carrying an episomal 41× *nmt* promoter-driven *HA-tpx1* gene (p123.41×), and processed by SDS-PAGE and analyzed by Western blot using antibodies against HA or Tpx1. Anti-tubulin was used as a loading control (Tub1). **b,c** Overexpression of OxyR cannot suppress the growth defects of cells lacking Tpx1, while catalase can. **b** Serial dilutions of YE cultures of strains 972 (WT), AD29 (WT + HA-OxyR), SG4 *(Δtpx1)*, AD36 *(Δtpx1* + HA-OxyR), and AD7 *(Δtpx1* + Ctt1) were spotted on YE agar plates and grown for 3 days at 30 °C under anaerobic or aerobic conditions. Both HA-OxyR and catalase were overexpressed to similar levels from integrative plasmids with the *sty1* promoter. **c** Growth of YE cultures of strains as in (**b**) was monitored by recording OD_600_ for a period of 30 h at 30 °C. **d** Determination of the steady-state levels of f4:5 intracellular H_2_O_2_ in cells lacking Tpx1 based on OxyR oxidation. MM cultures of AD36 (Δtpx1 expressing HA-OxyR) were treated or not with 0.2 mM f4:6 H_2_O_2_ for the times indicated, and TCA/AMS extracts were obtained and analyzed as described in Fig. 1b. The percentage of reduced (red.) and oxidized f4:7 (ox.) HA-OxyR in untreated. AMS 4-acetamido-4′-maleimidylstilbene-2,2′-disulfonic acid, MM minimal medium, ox. oxidized, red. reduced, SDS-PAGE sodium dodecyl sulphate-polyacrylamide gel electrophoresis, TCA trichloroacetic acid, WT wild type, YE rich medium, OD_600_ optical density at 600 nm

An experimental confirmation that OxyR reduction by Trx1 is not as effective as that of Tpx1 arises from the analysis of its capacity to scavenge H_2_O_2_. Thus, a H_2_O_2_ scavenging cycle requires fast oxidation of the peroxidase but also a fast reduction to restore its catalytic activity. As determined by Western blot analysis in Fig. 4a, the concentration of OxyR in our fission yeast system is comparable to that of Tpx1 (1.6 μM OxyR and 3.5 μM Tpx1 dimer). We analyzed whether OxyR could also contribute to basal H_2_O_2_ scavenging and therefore, suppress the phenotype of cells lacking Tpx1. While overexpression of a true H_2_O_2_ scavenger such as catalase can suppress the aerobic growth defects in liquid cultures (Fig. 4b) or on solid plates (Fig. 4c) of cells lacking Tpx1, overexpression of HA-OxyR cannot fully suppress those defects (Fig. 4b,c). We propose that the main difference between a H_2_O_2_ scavenger such as Tpx1 and a peroxide sensor such as OxyR lies in their reduction rates, which are much faster in the former.

Finally, we measured the basal percentage of oxidation of OxyR in a *Δtpx1* strain, to use it as an in vivo indicator of the intracellular steady-state levels of peroxides able to halt cell growth under aerobic conditions in cells lacking the main H_2_O_2_ scavenger, Tpx1. In this strain background, we interpret that the Trx system is fully available to recycle oxidized OxyR, since its main substrate, Tpx1, is absent. Assuming OxyR is 80% oxidized as shown in Fig. 4d at time 0, and using the correlation in Additional file 5: Figure S5, we estimated an intracellular concentration of H_2_O_2_ of 0.3 μM in basal conditions of strain *Δtpx1*. This level of H_2_O_2_ seems to be sufficient to slow down the growth rate, as observed in Fig. 4b, and to halt the growth of *S. pombe* on rich media solid plates (Fig. 4c).

### Kinetics of OxyR vs. Tpx1-Pap1 oxidation by peroxides: Tpx1 is more readily oxidized than OxyR, but Pap1 must compete with Trx1 for Tpx1 reduction

**Fig. 5:**
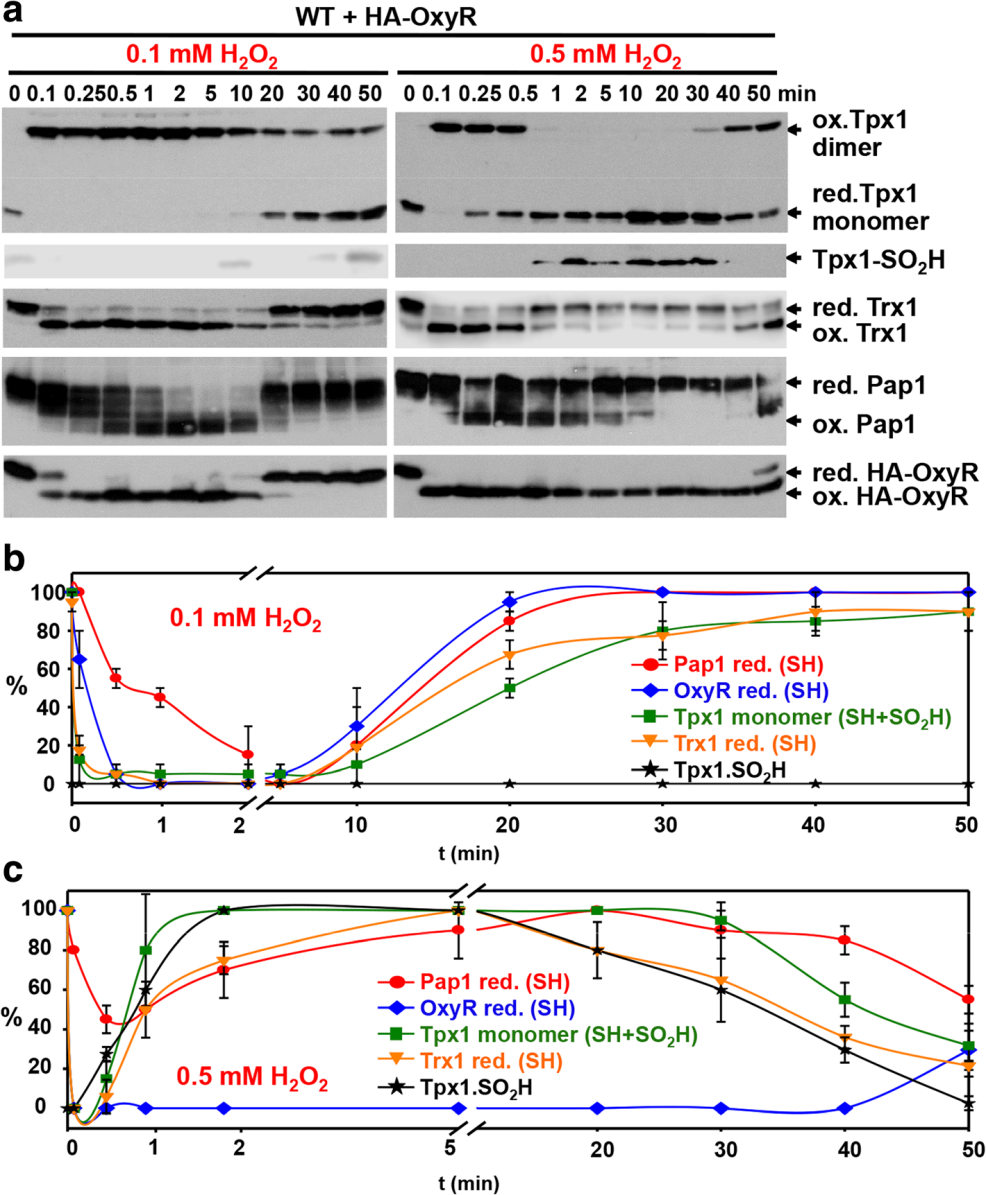
Kinetics of Tpx1, Trx1, Pap1, and HA-OxyR in wild-type cells upon sub-toxic and toxic concentrations of H_2_O_2_. **a** Cultures of strain AD29 (WT expressing HA-OxyR) were treated or not with 0.1 mM (left panels) or 0.5 mM (right panels) H_2_O_2_ for the indicated times, and protein extracts were obtained and processed as described in Fig. 1b, using antibodies against Tpx1 (ox. Tpx1 dimer and red. Tpx1 monomer), sulfinylated Tpx1 (Tpx1-SO_2_H), Trx1 (red. Trx1 and ox. Trx1), Pap1 (red. Pap1 and ox. Pap1), or HA (red. HA-OxyR and ox. HA-OxyR). **b** ,**c** Average of biological triplicates (**b**) or duplicates (**c**) of the experiment described in (**a**) are represented in curve graphs. Error bars (standard error of the mean) from the triplicates are shown. ox. oxidized, red. reduced, WT wild type

Once we established that Tpx1 and OxyR are present at comparable concentrations in the same cell type, *S. pombe*, and that both rely on the main thioredoxin, Trx1, for reduction, we analyzed the kinetics of oxidation of all the components of these two redox systems: Tpx1, Pap1, Trx1, and OxyR. We first applied an acute dose of 0.1 mM H_2_O_2_. This treatment does not affect the growth of wild-type cultures (Additional file 6: Figure S6a) but is known to activate the transcription factor Pap1 rapidly and transiently [13, 34]. As shown in Fig. 5a (left panels) and in Fig. 5b, Tpx1 and Trx1 oxidation occur almost immediately, with 50–90% oxidation only 5 s after stress imposition. Oxidation of Pap1, which is maximum at 120 s, occurs only when reduced Trx1 is exhausted [33]. OxyR oxidation is slightly slower than Tpx1-Trx1 oxidation. Oxidation of all these factors is maintained for 10–20 min. At this non-toxic concentration of extracellular peroxides, both systems, Tpx1-Pap1 and OxyR, behave similarly, at the level of both timing and duration of the active/oxidized stage. It is important to point out that at lower concentrations of extracellular peroxides such as 20 or 50 μM extracellular H_2_O_2_, Tpx1 oxidation occurs but Pap1 remains inactive, since reduced Tpx1 and Trx1 are not fully depleted from cells (Additional file 6: Figure S6b). At these concentrations, OxyR is already oxidized (Fig. 3 and Additional file 6: Figure S6b). Thus, OxyR senses concentrations of H_2_O_2_ lower than those sensed by Pap1, even though Tpx1 senses peroxides more than an order of magnitude earlier than OxyR.

Higher concentrations of H_2_O_2_, however, impinge on both types of cascades in different ways (Fig. 5a, right panel and Fig. 5c). We found that 0.5 mM extracellular peroxides exert toxic effects on fission yeast cells, reflected by the 4 h lag time on growth curves (Additional file 6: Figure S6a). Under these conditions, OxyR oxidation is even faster than before, with 100% oxidation only 5 s after stress imposition. OxyR is oxidized during the experiment, which is a reflection of the high intracellular levels of peroxides (Fig. 5c). In contrast, Tpx1, Trx1, and Pap1 undergo two waves of oxidation: (i) An initial transient oxidation lasts only 1–2 min, since after a sufficient number of cycles, Tpx1 is poisoned by these high concentrations of peroxides. Tpx1-SO_2_H accumulates and remains for 30–40 min (Fig. 5a, right panel and Fig. 5c; Tpx1-SO_2_H), probably until the levels of the Tpx1-SO_2_H reductase Srx1 have built up [12, 13]. (ii) Then, 40 min after stress imposition, a new wave of Tpx1-Pap1 oxidation appears, concomitant to the reduction of Tpx1-SO_2_H. From the time-course experiments, we can conclude that OxyR is a real sensor of H_2_O_2_, and remains oxidized until intracellular peroxide levels are below a certain threshold. The presence of reduced Trx1 cannot overcome the reaction rates of H_2_O_2_ with reduced OxyR, since with 0.5 mM peroxides, 100% of OxyR is oxidized from 2 to 40 min even though Trx1 is fully reduced. In contrast, the transcription factor Pap1 does not sense H_2_O_2_ levels. Rather, it has two requirements for a sustained oxidation: Tpx1 cycling and reduced Trx1 being exhausted.

**Fig. 6:**
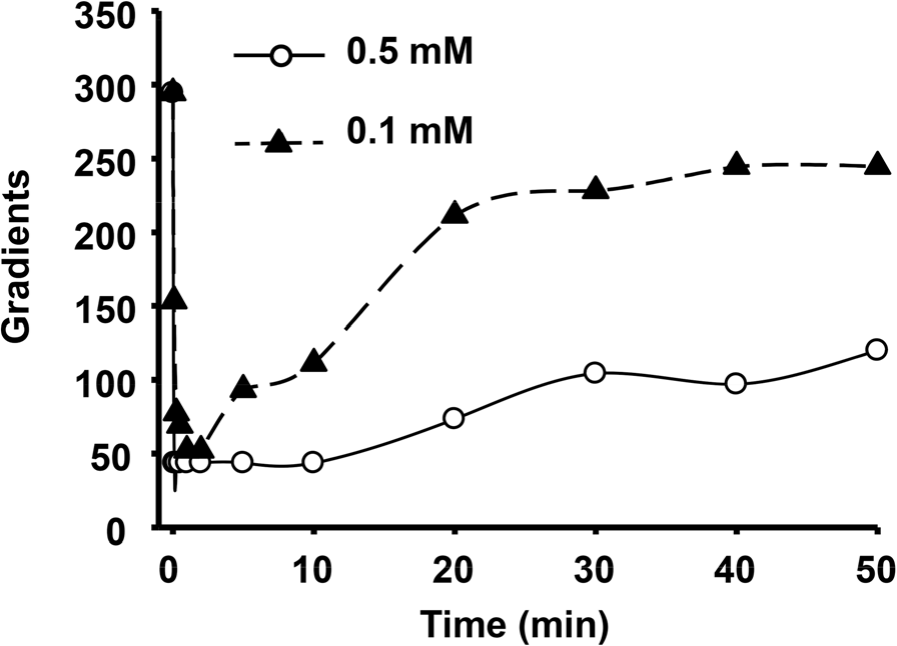
Theoretical temporal gradients of H_2_O_2_ in response to 0.1 (filled triangles) and 0.5 mM (open circles) H_2_O_2_. Gradients are estimated according to Eq. 4. *P*_*s*_ of 0.096 ± 0.034 μm s^− 1^ (*n* = 17) was determined in this work from experiments with OxyR oxidation, giving *k*_perm_ = 0.141 ± 0.051 s^− 1^ (*n* = 17). *k*_catabolism_ was estimated from the reduction levels of Tpx1 and catalase and rate constants of 1 × 10^7^ M^− 1^ s^− 1^ and 4.6 × 10^7^ M^− 1^ s^− 1^, respectively. When Tpx1 and catalase are 100% active in eliminating H_2_O_2_, *k*_catabolism_ = 41 s^− 1^, as indicated in Table 2

We used the experimentally determined Tpx1 oxidation ratios after applying sub-toxic and toxic doses of H_2_O_2_ (Fig. 5) to infer the temporal gradients of extracellular to intracellular peroxides using Eq. 4 (see Methods). As shown in Fig. 6, the gradients depend on the scavenging cellular capacity, and range from near 300:1 with maximum Tpx1 scavenging activity (i.e., when Tpx1 is in monomeric thiol form) to 40:1 (when Tpx1 is either fully oxidized to a disulfide or fully inactivated to the sulfinic acid form). These theoretical gradients are entirely compatible with the experimental gradients determined from OxyR oxidation previously, further validating our analysis. With the sub-toxic dose of 0.1 mM, the low gradient of 40 is temporary, with cells rapidly starting to recover the Tpx1-driven gradient of 300, while with the toxic dose of 0.5 mM the gradient collapse is much more sustained and does not recover even after 50 min. A higher extracellular concentration combined with a lower gradient is responsible for a higher intracellular concentration of H_2_O_2_ at this toxic level.

## Discussion

Full activation of antioxidant transcription factors by moderate fluctuations of H_2_O_2_ occurs through oxidation of Cys residues, and two sensing models have been proposed. OxyR is a one-component redox cascade, and eukaryotic Prx (Tpx1)-transcription factor (Pap1) is a two-component redox relay. We have combined experimentally obtained data and applied equations to measure H_2_O_2_ gradients through biological membranes, to calculate intracellular peroxide levels involved in signaling and in toxicity, and to define the two modes of action of the two sensing modules. While OxyR is a direct H_2_O_2_ sensor, Pap1 oxidation follows two premises: (i) It is oxidized by Tpx1 only when reduced Trx1 is transiently depleted and Tpx1 is no longer acting as a peroxide scavenger. (ii) At high H_2_O_2_ doses, the Tpx1 oxidation cycle can collapse due to Tpx1.SO_2_H formation, Pap1 becoming insensitive to peroxides until Srx1 facilitates sulfinic acid reduction.

Only OxyR and Tpx1 contain true Cys redox switches, and therefore, are able to detect mild fluctuations of peroxides. Both display fast second-order oxidation reaction rates, with that for Tpx1 being one or two orders of magnitude higher than that for OxyR. However, the Tpx1 reduction/recycling kinetics are also extremely fast, while OxyR reduction is not, at least once expressed in fission yeast. The ratio between the rate of oxidation and the rate of reduction of a thiol protein is very important in defining its response to H_2_O_2_ [26]. In the present case, we observed that the reduction rate of Tpx1 is more than 20 times higher than OxyR reduction. As a consequence, Tpx1 can efficiently scavenge peroxides, since reduced Tpx1 is readily available after each oxidation cycle, while OxyR cannot directly act as a peroxide scavenger, even when overexpressed in fission yeast.

**Fig. 7:**
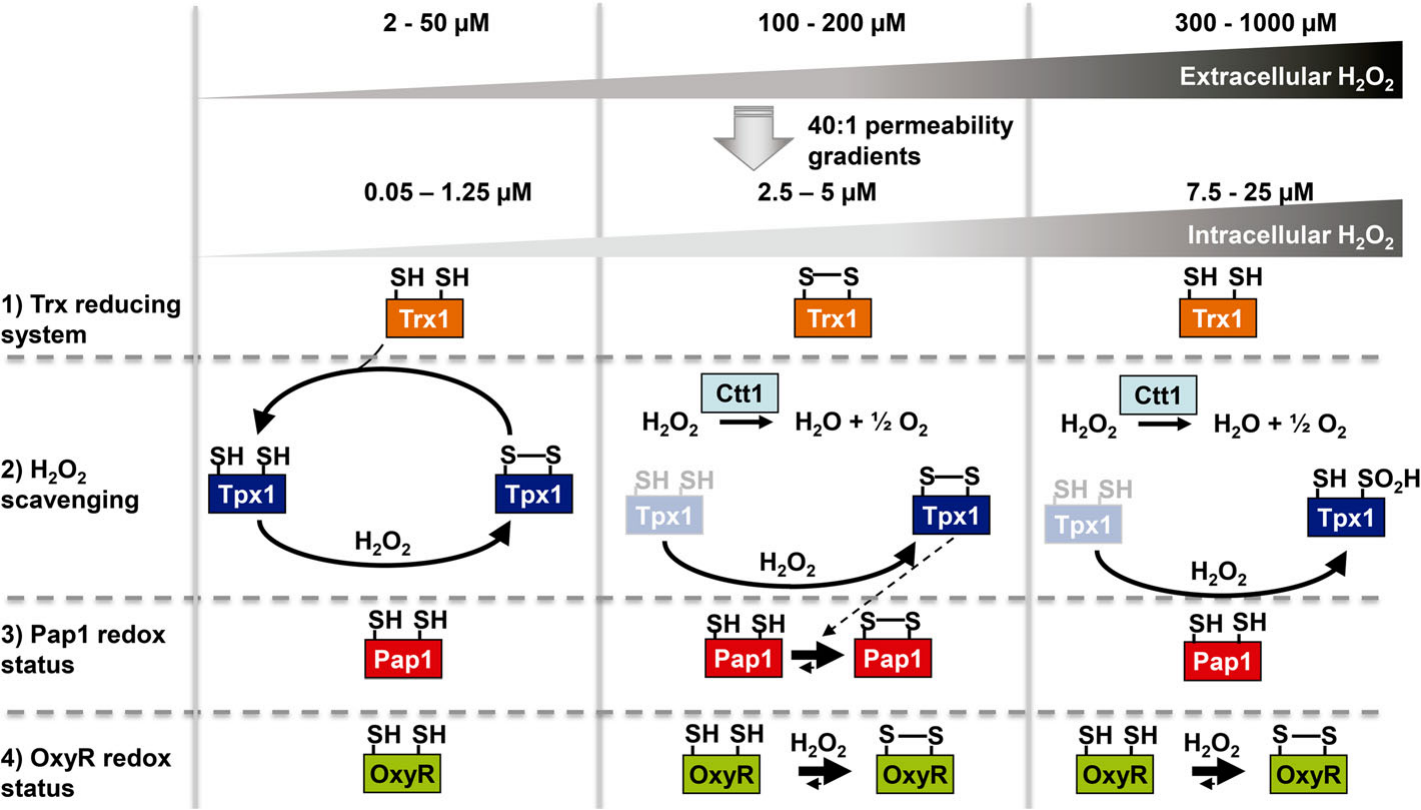
Model of H_2_O_2_ scavenging and sensing at different oxidant concentrations. From our experimental data and applied biochemical equations, we propose three scenarios depending on the extracellular peroxide levels. A permeability gradient of 40:1 (extracellular to intracellular) is indicated, with further gradients achieved only when Tpx1 is fully active. At 2–50 μM extracellular peroxides, Tpx1 acts as the only H_2_O_2_ scavenger, while at sub-toxic doses of extracellular peroxide (100–200 μM), both Tpx1 and Ctt1 act as scavengers. Finally, at toxic levels of extracellular peroxides (300–1000 μM), the main intracellular scavenger of peroxides is Ctt1. Regarding signaling, while OxyR senses peroxides at both sub-toxic (100–200 μM) and toxic (0.3–1 mM) doses, Pap1 is active only at the intermediate concentrations, due to Tpx1 acting as a scavenger at the lowest doses thanks to Trx1-mediated recycling, and Tpx1 being over-oxidized to the sulfinic acid form at the highest H_2_O_2_ doses

Another consequence of the fast reduction of Tpx1 is the induction of retroactivity by the consumption of oxidized Tpx1, diverting it from Pap1 oxidation. In biological systems, retroactivity can be defined as a flow of matter between two modules in which the downstream module affects the dynamic behavior of the upstream module [35]. In the present case, the diversion of Tpx1 oxidized moieties from Pap1 oxidation strongly impacts the response of Tpx1/Pap1 to H_2_O_2_ fluctuations. Due to the intrinsic properties of both signaling cascades, similar sub-toxic concentrations of peroxides, of the order of 100 μM extracellular H_2_O_2_, end up fully activating both transcription factors, OxyR and Pap1, even though Tpx1 senses peroxides more than 10 times faster than OxyR (Fig. 7, center panel). In fact, OxyR can be oxidized at 50 μM (Fig. 3 and Additional file 6: Figure S6b). This important observation indicates that in the two-component redox relay, a kinetic barrier exists when relaying the oxidation from the first component for the second component. This barrier can be very high if the first component is reduced very fast, and in such cases the signal is relayed only when the reducing partner of the first component is also oxidized. This Tpx1-Pap1 cellular design, in which the same protein, a Prx, shares H_2_O_2_ scavenging and signaling roles, links aerobic metabolism to the activation of antioxidant cascades, since activation occurs only once the cell’s reducing power is saturated (i.e., the reduced cofactor is exhausted and oxidized Trx1 cannot be recycled). Thus, in the Tpx1-Pap1 design, H_2_O_2_ sensing and the redox state the cell are strongly connected by the fast rate of Tpx1 reduction and thus, Tpx1-Pap1 can be defined as a bifunctional module that responds to both H_2_O_2_ fluctuations and the redox state of the cell as defined by the Trx1 oxidation state.

In contrast, the relatively fast oxidation rate of OxyR combined with its slow reduction kinetics eases the sustained oxidation of the transcription factor upon mild to severe H_2_O_2_ threats, so that OxyR is maintained in the active form long enough to exert its antioxidant role as a transcription factor. We propose that OxyR is a true H_2_O_2_ sensor and redox transducer. It was proposed by Aslund and colleagues that the oxidation of OxyR in *Escherichia coli* is constitutive in certain strain backgrounds, even in the absence of added oxidants, since the oxidation and activity of OxyR was elevated in mutants of the Trx and Grx systems. However, we show here that the thiol switch in OxyR, Cys199, does not detect disturbances in the fission yeast Grx system. Individual *trx1* or *trx3* deletion only affects the speed of OxyR reduction after stress imposition, but not the basal redox state of OxyR. Thus, in the OxyR design, the response to H_2_O_2_ fluctuations is partially insulated from the redox state of the cell, reinforcing our proposal that OxyR oxidation acts as a module whose single function is to sense H_2_O_2_.

Signaling upon high toxic levels of peroxides impinges very differently in both antioxidant transcription factors (Fig. 7, right panel). While OxyR stays fully oxidized for longer than after mild oxidative stress, Pap1 responds in a dual kinetic fashion upon these Tpx1-inactivating H_2_O_2_ doses. Thus, Pap1 suffers two waves of oxidation, the former being very fast (lasting only 2 min after 0.5 mM extracellular peroxides) and the second being more sustained (starting 30–40 min after stress imposition). Only this second wave lasts enough to allow oxidized/active Pap1 to engage the antioxidant gene expression program. This delayed activation of the transcription factor is caused by the temporal inactivation of Tpx1 by high doses of peroxides, which accumulates with its peroxidatic Cys in the sulfinic acid form. Reduction of Tpx1.SO_2_H depends on Srx1, which is overexpressed in response to the activation of a second redox pathway, governed by Sty1 and Atf1 [12, 13]. Thus, the Prx-driven activation of transcription factors by H_2_O_2_ implies than an alternative cascade controls the antioxidant adaptation to high doses of peroxides, in this case the MAP kinase Sty1 cascade.

Combining our experimental data with biochemical equations, we have determined the threshold of intracellular H_2_O_2_ concentration able to halt aerobic growth: 0.3 μM. These steady-state doses, detected in liquid cultures of *Δtpx1* cells, are sufficient to seriously jeopardize aerobic growth. Tpx1, with its exquisite sensitivity for its substrate, is fully responsible for decreasing those steady-state levels during aerobic metabolism, and therefore Tpx1 is essential only when cells are grown in the presence of oxygen [23]. The main H_2_O_2_ scavenger upon toxic doses of extracellular peroxides is catalase, since Tpx1 is over-oxidized at its peroxidatic Cys to SO_2_H (Fig. 7, right panel). However, at intermediate, sub-toxic doses of peroxides, those fully activating the Pap1 cascade, both catalase and Tpx1 contribute to H_2_O_2_ scavenging, since disulfide accumulation in Tpx1 can temporarily block its peroxidase capacity (Fig. 7, center panel). In fact, we detect Tpx1.SO_2_H formation after mild 100 μM H_2_O_2_ stress in *Δctt1* cells (Additional file 6: Figure S6c), what suggests that intracellular levels of peroxides are higher than in a wild-type background.

Applying mathematical equations to the experimental data on OxyR oxidation and on extracellular H_2_O_2_ consumption rates, we have also determined that the gradient through *S. pombe* membranes is around 40:1, but that intracellular H_2_O_2_ scavenging activities can enhance the gradient up to 300:1. Using Tpx1 oxidation on different concentrations of applied peroxides (Fig. 5), we calculated the transient extracellular-to-intracellular gradients, which vary largely depending on the applied H_2_O_2_ levels and on the corresponding cellular scavenging activities. Interestingly, the calculated gradients closely follow the oxidation profile of OxyR, further strengthening the H_2_O_2_ sensor role of this thiol protein.

In conclusion, our study compared the two main models of H_2_O_2_ sensing and transduction. While OxyR is a real on/off-switch transcription factor and triggers an antioxidant response for as long as peroxides are over a threshold, the Tpx1-Pap1 relay system constitutes a sophisticated sensing module. Tpx1 operates during aerobic metabolism as a H_2_O_2_ scavenger, and only when the cellular recycling power is exhausted, is Pap1 activated. Higher, toxic doses of peroxides poison the two-component system and therefore, an alternative signal transduction cascade, such the Sty1-Atf1 pathway, is required.

## Conclusions

Here we report on the characterization of two well-established sensor modules of H_2_O_2_, OxyR and Tpx1-Pap1. Applying mathematical equations to the experimental kinetic data, we have defined that (i) 0.3 μM intracellular H_2_O_2_ is a toxic threshold capable of halting cell growth; (ii) the gradient of extracellular to intracellular peroxides through fission yeast membranes is around 40:1, but that intracellular H_2_O_2_ scavenging activities can enhance this gradient up to 300:1; and (iii) while Tpx1 is in essence a H_2_O_2_ scavenger due to fast oxidation and reduction kinetics, OxyR is a true H_2_O_2_ sensor but not a scavenger, since it cannot be reduced as fast as it is oxidized.

## Methods

### Growth conditions, genetic manipulation, and strains

Cells were grown in rich medium (YE) or minimal medium (MM) at 30 °C as described previously [36]. The origins and genotypes of strains used in this study are outlined in Additional file 7: Table S1. Most of them were constructed by standard genetic methods. Plasmid p490', containing the *HA-oxyR* gene under the control of the constitutive *sty1* promoter, was linearized and integrated by homologous recombination at the *leu1–32* locus of different strain backgrounds. Some strains were obtained by crossing. To overexpress catalase, we cloned the *ctt1* gene into pREP.42× [37] to yield plasmid p418.42×. The *nmt* promoter from plasmid p418.42× was replaced with a constitutive *sty1* promoter from plasmid p386' [38], to yield the episomal plasmid p464. To quantify HA-OxyR in fission yeast relative to another HA-containing protein for which we have a polyclonal antibody, we used episomal plasmid p123.41×, expressing HA-Tpx1 under the control of the *nmt41* promoter [13].

### Trichloroacetic acid extracts and immuno-blot analysis

Modified trichloroacetic acid (TCA) extracts were prepared by blocking thiols with 4-acetamido-4′-maleimidylstilbene-2,2′-disulfonic acid (AMS) and separated in non-reducing denaturing electrophoresis as previously described [13] with the following modifications. AMS is a bulky alkylating agent that alkylates cysteines in the thiol form only, which have a net molecular weight of ~0.5 kDa for each moiety of AMS incorporated. Acetone-washed TCA cell pellets corresponding to 5 ml of cultures (5 × 10^7^ cells) were resuspended in 50 μl of 2.5 mM AMS (from a 25 mM stock) in 200 mM Tris-HCl (pH 7.5), 1 mM ethylenediaminetetraacetic acid (EDTA), and 1% sodium dodecyl sulphate (SDS). Samples were incubated for 2 h at 37 °C. Extracts were separated by non-reducing SDS-polyacrylamide gel electrophoresis (SDS-PAGE) and analyzed by Western blot. HA-OxyR, Tpx1, Tpx1.SO_2_H, Pap1, and Trx1 were immuno-detected with monoclonal house-made anti-HA antibodies, or with anti-Tpx1 [23], anti-peroxiredoxin-SO3 antibody (LabFrontier, Seoul, South Korea), anti-Pap1 [13], and anti-Trx1 [39] polyclonal antibodies, respectively. Anti-Sty1 polyclonal antibody [23] was used as the loading control. All the Western blots and experiments described in the manuscript have been biologically replicated with almost no variation, as shown in Additional file 8: Table S7. Relative quantification of protein levels in Western blots was performed by scanning membranes with a Licor 3600 CDigit Blot Scanner (Licor Inc., USA) and using the Image Studio™ 4.0 software. Quantifications of the Western blots used to create Fig. 3, Fig. 5b, c, Fig. 6 and Tables 3 and 4 are included in Additional file 9. To calculate the intracellular concentration of HA-OxyR in fission yeast, we obtained TCA extracts of wild-type cells expressing or not HA-OxyR, and of *∆tpx1* expressing HA-Tpx1 from a plasmid. We normalized the protein extracts with commercial anti-tubulin antibodies, and we determined the relative levels of HA-Tpx1 using anti-Tpx1 antibodies. We used the same extracts to compare the expression levels of HA-OxyR using in-house anti-HA antibodies. Knowing that the intracellular concentration of Tpx1 is around 7 μM [25, 40], we determined that the intracellular concentration of the HA-OxyR monomer expressed in fission yeast is 1.6 ± 0.3 μM.

### Oxygen sensitivity assay on solid plates

To measure survival on solid plates, *S. pombe* strains were grown, diluted, and spotted on YE plates, which were incubated at 30 °C for 2 or 3 days. To grow cells in solid media in an anaerobic environment, we incubated the plates at 30 °C in a tightly sealed plastic bag containing a water-activated Anaerocult A sachet (Merck, Darmstadt, Germany) [23] or in a nitrogen-filled anaerobic chamber (Forma Anaerobic System, Thermo Electron Corp.).

### Growth curves

Yeast cells were grown in YE or MM from an initial OD_600_ of 0.2 or 0.5, as indicated, in 96-well plates. Cell growth was monitored using an assay based on automatic measurements of optical densities, as previously described [41].

### H_2_O_2_ scavenging by whole cells

After the addition of the indicated concentrations of H_2_O_2_ to MM cultures of strains at an OD_600_ of 0.5, 1-ml aliquots were taken at different time points, and stopped by the addition of 110 μl of TCA 100%. Samples were then centrifuged to eliminate cells and 100 μl of the supernatant was used to determine the remaining H_2_O_2_ concentration. This was achieved by the addition of 27 μl of 10 mM ferrous ammonium sulfate and 13.5 μl of 2.5 M potassium thiocyanate. The red ferricthiocyanate complex, which appears because of the oxidation of Fe(II) by H_2_O_2_, was quantified by measuring the OD at 480 nm. It was compared to H_2_O_2_ standards, ranging from 10 to 100 μM H_2_O_2_.

### Experimental determination of concentration gradients between extracellular and intracellular H_2_O_2_ based on OxyR oxidation kinetics and extracellular consumption of H_2_O_2_

Two methods were used to estimate gradients from experimental data. In the first, the profile of oxidation of OxyR was fitted to [42]:

1 $$ {\left.{\mathrm{Target}}_{\mathrm{rd}}\right|}_t=\frac{k_{\mathrm{switch}\hbox{-} \mathrm{off}}}{k_{\mathrm{switch}\hbox{-} \mathrm{off}}+{k}_{\mathrm{activation}}}+{e}^{-\left({k}_{\mathrm{switch}\hbox{-} \mathrm{off}}+{k}_{\mathrm{activation}}\right)\times t}\times \left({\left.{\mathrm{Target}}_{\mathrm{rd}}\right|}_0-\frac{k_{\mathrm{switch}\hbox{-} \mathrm{off}}}{k_{\mathrm{switch}\hbox{-} \mathrm{off}}+{k}_{\mathrm{activation}}}\right). $$

In Eq. 1, Target_rd_|_*t*_ refers to the fraction of the thiol protein in the reduced form observed at time *t*. *k*_activation_ and *k*_switch-off_ are pseudo-first-order rate constants for the oxidation and reduction of the thiol protein. *k*_activation_ can be written as [H_2_O_2_] × *k*_target + H2O2_ with [H_2_O_2_] being the H_2_O_2_ concentration attained in the vicinity of the thiol protein and *k*_target + H2O2_ being the second-order rate constant for the reaction between the thiol protein and H_2_O_2_. Finally, Target_rd_|_0_, is the fraction of the thiol protein in the reduced form observed at time 0. *k*_activation_ and *k*_switch-off_ were estimated from non-linear fits to the experimental data. From *k*_activation_, the intracellular H_2_O_2_ concentration attained during the experiment was calculated using the published value *k*_target + H2O2_ = 1.4 × 10^5^ M^− 1^ s^− 1^ for OxyR and therefore, by inputting the known experimental extracellular H_2_O_2_ concentration, the gradient between the extracellular and the intracellular concentrations of H_2_O_2_ was estimated.

Alternatively, experimental H_2_O_2_ concentration gradients were determined from the ratio between the rate of removal of intracellular H_2_O_2_ and the rate of removal of extracellular H_2_O_2_. The removal of extracellular H_2_O_2_ is obtained from the pseudo-first-order rate constant (*k*_cons_), which characterizes the kinetics of H_2_O_2_ consumption by intact cells. *k*_cons_ was determined by fitting the H_2_O_2_ consumption profiles shown in Additional file 4: Figure S4 to an exponential decay (Table 1). The intracellular removal of H_2_O_2_ is estimated from the published concentrations of Tpx1 and Ctt1 and respective second-order rate constants for the reaction with H_2_O_2_ (see Table 2).

Thus, these two methods are fully independent of each other, as they rely on different experimental measurements and published values.

### Steady-state analysis of OxyR and Tpx1 oxidation levels

At steady-state, the rate of OxyR oxidation by H_2_O_2_ matches the rate of OxyR reduction by Trx systems, or

2a $$ {k}_{H 2O 2\_ OxyR}\times \left[{\mathrm{H}}_2{\mathrm{O}}_2\right]\times {\left[\mathrm{OxyR}\right]}_{\mathrm{rd}}={k}_{\mathrm{switch}-\mathrm{off}}\times {\left[\mathrm{OxyR}\right]}_{\mathrm{ox}}, $$

2b $$ {k}_{H 2O 2\_ OxyR}\times \left[{\mathrm{H}}_2{\mathrm{O}}_2\right]\times {\left[\mathrm{OxyR}\right]}_{\mathrm{rd}}=\left({k}_{Trx1\_ OxyR}\times {\left[\mathrm{Trx}1\right]}_{\mathrm{rd}}+{k}_{Trx3\_ OxyR}\times {\left[\mathrm{Trx}3\right]}_{\mathrm{rd}}\ \right)\times {\left[\mathrm{OxyR}\right]}_{\mathrm{ox}}, $$

where *k*_*H2O2_OxyR*_ is the second-order rate constant for the oxidation of OxyR by H_2_O_2_. *k*_switch-off_ lumps all activities that are able to reduce OxyR, and here, only those originating in Trx1 and Trx3 were included. *k*_*Trx1_OxyR*_ and *k*_*Trx3_OxyR*_ are the second-order rate constants for the reduction of oxidized OxyR by Trx1 and Trx3, respectively.

Likewise, in the steady state, the rate of Tpx1 oxidation by H_2_O_2_ matches the rate of Tpx1 reduction by Trx systems, or

3a $$ {k}_{H 2O 2\_ Tpx1}\times \left[{\mathrm{H}}_2{\mathrm{O}}_2\right]\times {\left[\mathrm{Tpx}1\right]}_{\mathrm{rd}}={k}_{\mathrm{switch}-\mathrm{off}}\times {\left[\mathrm{Tpx}1\right]}_{\mathrm{ox}}, $$

3b $$ {k}_{H 2O 2\_ Tpx1}\times \left[{\mathrm{H}}_2{\mathrm{O}}_2\right]\times {\left[\mathrm{Tpx}1\right]}_{\mathrm{rd}}={k}_{Trx\_ Tpx1}\times {\left[\mathrm{Trx}\right]}_{\mathrm{rd}}\times {\left[\mathrm{Tpx}1\right]}_{\mathrm{ox}}, $$

where *k*_*H2O2_Tpx1*_ is the second-order rate constant for the oxidation of Tpx1 by H_2_O_2_, *k*_*Trx_Tpx1*_ is the second-order rate constant for the reduction of oxidized Tpx1 by a thioredoxin, and *k*_switch-off_ is equal to *k*_*Trx_Tpx1*_ × [Trx]_rd_.

### Theoretical calculation of gradients

The theoretical gradients were estimated as:

4 $$ \frac{{\left[{\mathrm{H}}_2{\mathrm{O}}_2\right]}_{\mathrm{extracellular}}}{{\left[{\mathrm{H}}_2{\mathrm{O}}_2\right]}_{\mathrm{intracellular}}}=\frac{k_{\mathrm{perm}}+{k}_{\mathrm{catabolism}}}{k_{\mathrm{perm}}}, $$

where *k*_catabolism_ is the overall pseudo-first-order rate constant for the intracellular consumption of H_2_O_2_ and *k*_perm_ is the pseudo-first-order rate constant for the permeation of H_2_O_2_ across the plasma membrane. The intracellular consumption of H_2_O_2_ was calculated based on published values of kinetics and proteins levels, as shown in Table 2, and *k*_perm_ was calculated with

5 $$ {k}_{\mathrm{perm}}={P}_s\frac{\mathrm{Area}}{\mathrm{Volume}}, $$

where *P*_*s*_ is the permeability coefficient for H_2_O_2_ across the plasma membrane and the area and volume are the geometrical characteristics of the cell. An area and volume of 99 μm^2^ and 146 μm^3^, respectively, were calculated by assuming that a *S. pombe* cell is a cylinder with a diameter of 3 μm and a height of 14 μm [43]. *P*_*s*_ was determined from [44]:

6 $$ {P}_s=\frac{k_{\mathrm{catabolism}}\times R}{\frac{\mathrm{Area}}{\mathrm{Volume}}\left(1-R\right)}, $$

where *R* is the ratio between the intracellular and extracellular H_2_O_2_ concentrations.

## Additional files

Additional file 1: **Figure S1.** Expression of bacterial OxyR in Prx, Trx, and Grx mutants. **a** Prxs-independent oxidation of OxyR in fission yeast. Cultures of strains AD29 (WT), AD36 (*Δtpx1*), and AD58 (*Δtpx1 Δgpx1 Δpmp20 Δbcp1*), carrying an integrative *sty1* promoter-driven *HA-oxyR* gene, were treated or not with 0.2 mM H_2_O_2_ for the times indicated. TCA extracts were analyzed as in Fig. 2a. **b** Grx1 is not responsible for OxyR reduction in fission yeast. Cultures of strains AD29 (WT), AD59 (*Δgrx1*), and AD106 (*Δpgr1*), carrying an integrative *sty1* promoter-driven *HA-oxyR* gene, were treated or not with 0.2 mM H_2_O_2_ for the times indicated. TCA extracts were analyzed as in Fig. 2a. (PDF 83 kb)

Additional file 2: **Figure S2.** Kinetics of OxyR and Tpx1 oxidation. **a** In cells deficient in Tpx1 or Trx1, HA-OxyR oxidizes at low concentrations of peroxides. Aerobic or anaerobic cultures of strains AD29 (WT), AD61 (*Δtrx1*), and AD36 (*Δtpx1*) carrying an integrative *sty1* promoter-driven *HA-oxyR* gene were treated or not with the indicated concentrations of H_2_O_2_ for the times indicated. TCA extracts were analyzed as in Fig. 2a. **b** Cultures of strain AD29 (WT) were treated or not with 20, 50, or 100 μM H_2_O_2_ for the times indicated. TCA extracts were analyzed as in Fig. 2a, using antibodies against Tpx1 (ox. Tpx1 dimer is the upper band in the panels; red. Tpx1 monomer is the lower band in the panels). (PDF 476 kb)

Additional file 3: **Figure S3.** Determination of gradients between extracellular and intracellular H_2_O_2_ concentrations. Gradients are obtained from the non-linear fitting of Eq. 1 (dashed lines) to OxyR oxidation measured experimentally (filled squares) after adding the indicated concentration of H_2_O_2_. Experiments were done under aerobic conditions with wild-type, *Δtrx1*, or *Δtrx3* strains (**a**) or under anaerobic conditions in the *Δtpx1* strain (**b**). (PDF 150 kb)

Additional file 4: **Figure S4.** Both Tpx1 and Ctt1 participate in peroxide scavenging at sub-toxic doses of H_2_O_2_, while only catalase acts as a scavenger of the toxic ones. The concentration of the remaining extracellular peroxides of MM cultures of strains 972 (WT), SG4 (*Δtpx1*), EP198 (*Δctt1*), and SG267 (*Δtpx1 Δctt1*), treated with 0.1 or 0.5 mM H_2_O_2_, was determined at the times indicated with a colorimetric reaction (see Methods). (PDF 111 kb)

Additional file 5: **Figure S5.** OxyR as a sensor of intracellular H_2_O_2_ concentrations. The relation between intracellular H_2_O_2_ concentration and OxyR oxidation levels observed at steady state after addition of H_2_O_2_ to the WT (filled squares), *Δtrx1* (filled diamonds), or *Δtrx3* (filled circles) strains is plotted according to the data shown in Table 1 in the main text. (PDF 17 kb)

Additional file 6: **Figure S6.** At non-toxic concentrations of extracellular peroxides, both systems, Pap1-Tpx1 and OxyR, behave similarly. **a** Growth curves of strain AD29 (WT + HA-OxyR), treated or not with 0.1 mM or 0.5 mM H_2_O_2_, were recorded for 20 h. **b** Cultures of strain AD29 (WT + HA-OxyR) were treated or not with the indicated concentrations of H_2_O_2_ for the times indicated, and protein extracts were obtained and processed as described in Fig. 2a, using antibodies against HA (red. HA-OxyR and ox. HA-OxyR), Tpx1 (ox. Tpx1 dimer and red. Tpx1 monomer), Pap1 (red. Pap1 and ox. Pap1), or Trx1 (red. Trx1 and ox. Trx1). **c** Catalase acts as a scavenger at 100 μM, but not at 50 μM H_2_O_2_. Cultures of strains AD29 (WT) and AD163 (*Δctt1*), carrying an integrative *sty1* promoter-driven *HA-oxyR* gene, were treated or not with 50 or 100 μM H_2_O_2_ for the times indicated, and protein extracts were obtained and processed as described in Fig. 2a, using antibodies against Tpx1 (ox. Tpx1 dimer and red. Tpx1 monomer), sulfinylated Prx (Tpx1-SO_2_H), Trx1 (red. Trx1 and ox. Trx1), Pap1 (red. Pap1 and ox. Pap1), or HA (red. HA-OxyR and ox. HA-OxyR). (PDF 281 kb)

Additional file 7: **Table S1.** Strains used in this study. (PDF 10 kb)

Additional file 8: **Figure S7.** Biological replicates of main figures. (PDF 1383 kb)

Additional file 9: **Tables S2** to **S7.** Quantification of Western blots. (XLSX 48 kb)
