# Supplementary figures and images for: Using in vivo oxidation status of one- and two-component redox relays to determine H2O2 levels linked to signaling and toxicity
Source: BMC Biol. 2018 Jun 1;16:61. doi: 10.1186/s12915-018-0523-6 (PMC5984441; doi:10.1186/s12915-018-0523-6)

**a**

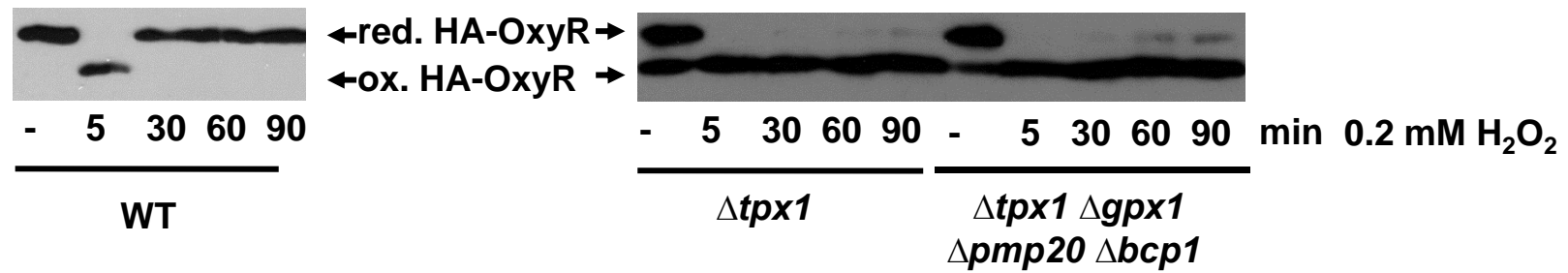

**b**

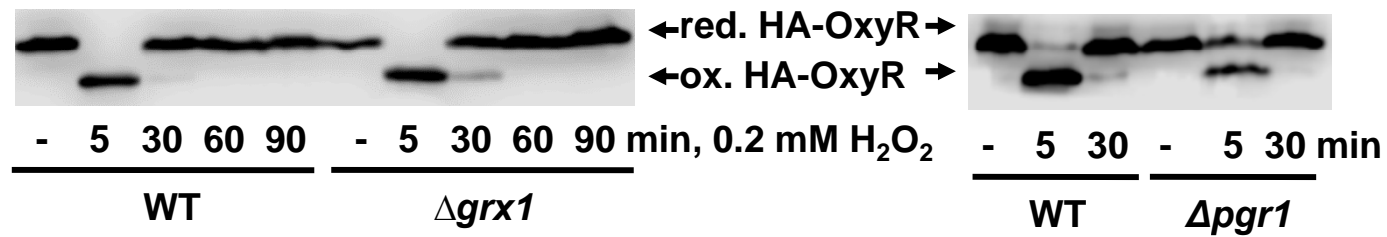

Domènech et al. Additional file 1: Figure S1

Supplement: Supplementary file 1 — Figure S1. Expression of bacterial OxyR in Prx, Trx, and Grx mutants. a Prxs-independent oxidation of OxyR in fission yeast. Cultures of strains AD29 (WT), AD36 (Δtpx1), and AD58 (Δtpx1 Δgpx1 Δpmp20 Δbcp1), carrying an integrative sty1 promoter-driven HA-oxyR gene, were treated or not with 0.2 mM H2O2 for the times indicated. TCA extracts were analyzed as in Fig. 2a. b Grx1 is not responsible for OxyR reduction in fission yeast. Cultures of strains AD29 (WT), AD59 (Δgrx1), and AD106 (Δpgr1), carrying an integrative sty1 promoter-driven HA-oxyR gene, were treated or not with 0.2 mM H2O2 for the times indicated. TCA extracts were analyzed as in Fig. 2a. (PDF 83 kb) [file 12915_2018_523_MOESM1_ESM.pdf]

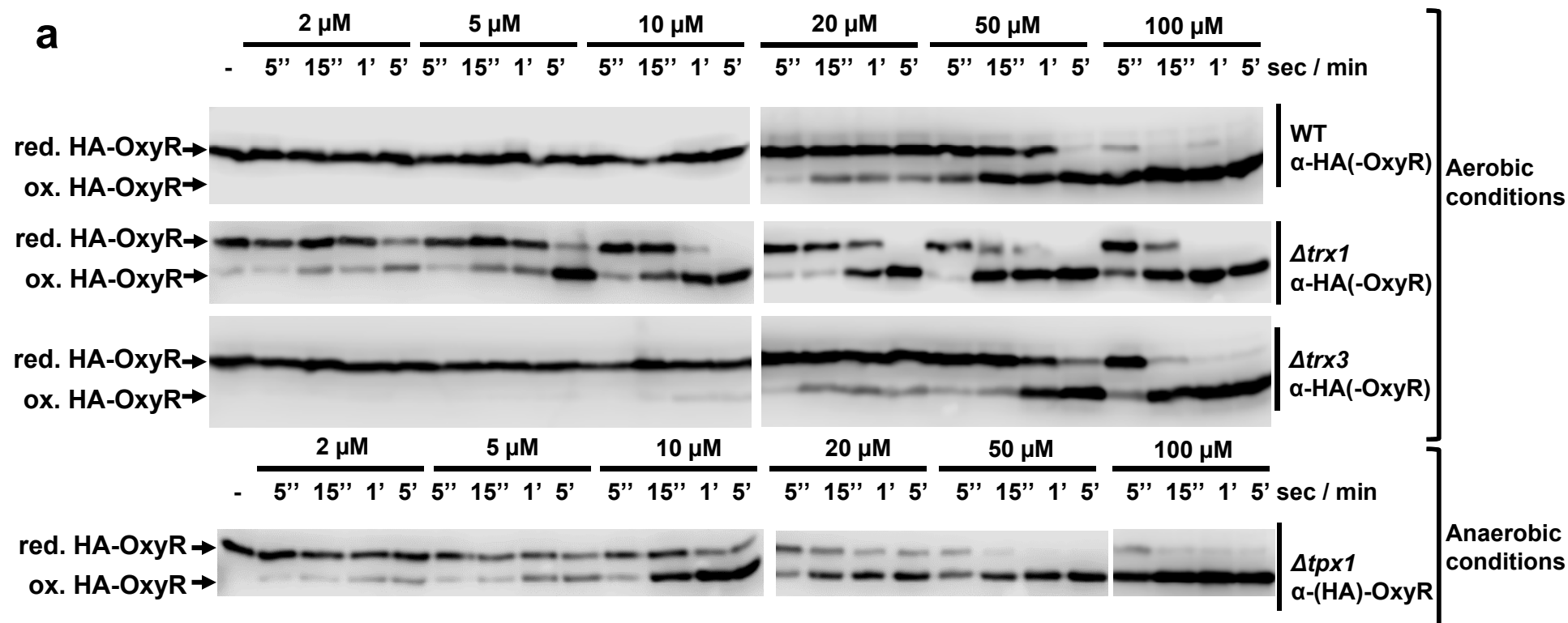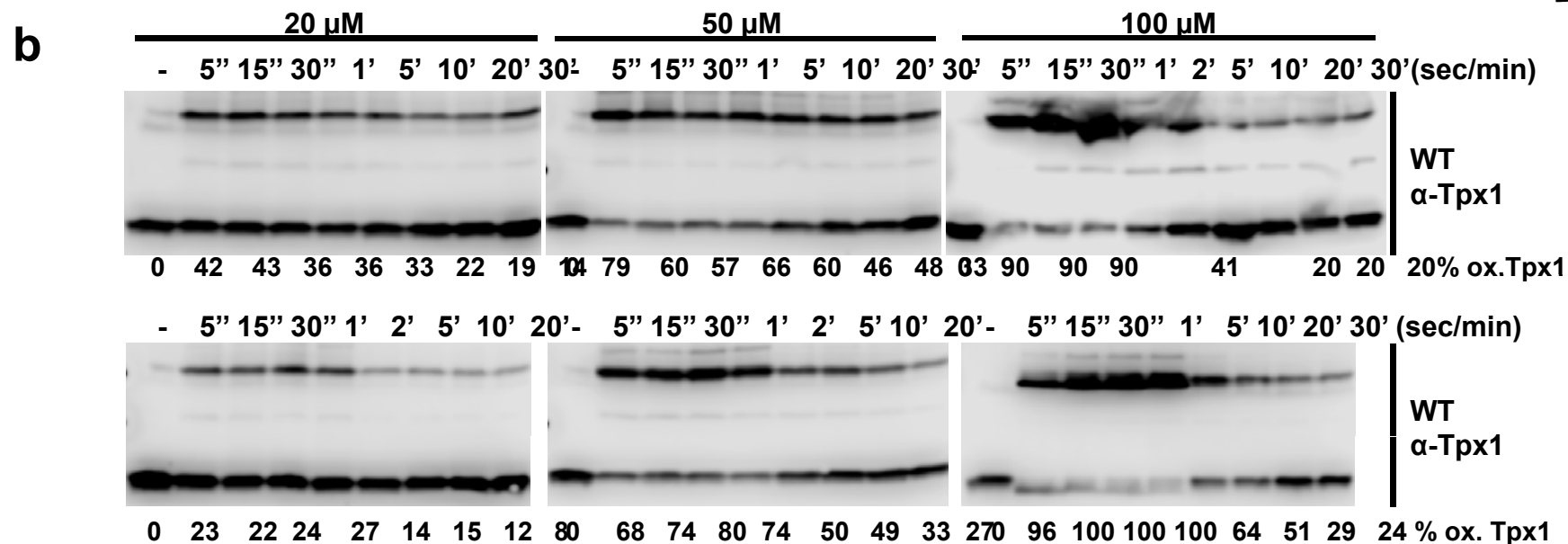

Supplement: Supplementary file 2 — Figure S2. Kinetics of OxyR and Tpx1 oxidation. a In cells deficient in Tpx1 or Trx1, HA-OxyR oxidizes at low concentrations of peroxides. Aerobic or anaerobic cultures of strains AD29 (WT), AD61 (Δtrx1), and AD36 (Δtpx1) carrying an integrative sty1 promoter-driven HA-oxyR gene were treated or not with the indicated concentrations of H2O2 for the times indicated. TCA extracts were analyzed as in Fig. 2a. b Cultures of strain AD29 (WT) were treated or not with 20, 50, or 100 μM H2O2 for the times indicated. TCA extracts were analyzed as in Fig. 2a, using antibodies against Tpx1 (ox. Tpx1 dimer is the upper band in the panels; red. Tpx1 monomer is the lower band in the panels). (PDF 476 kb) [file 12915_2018_523_MOESM2_ESM.pdf]

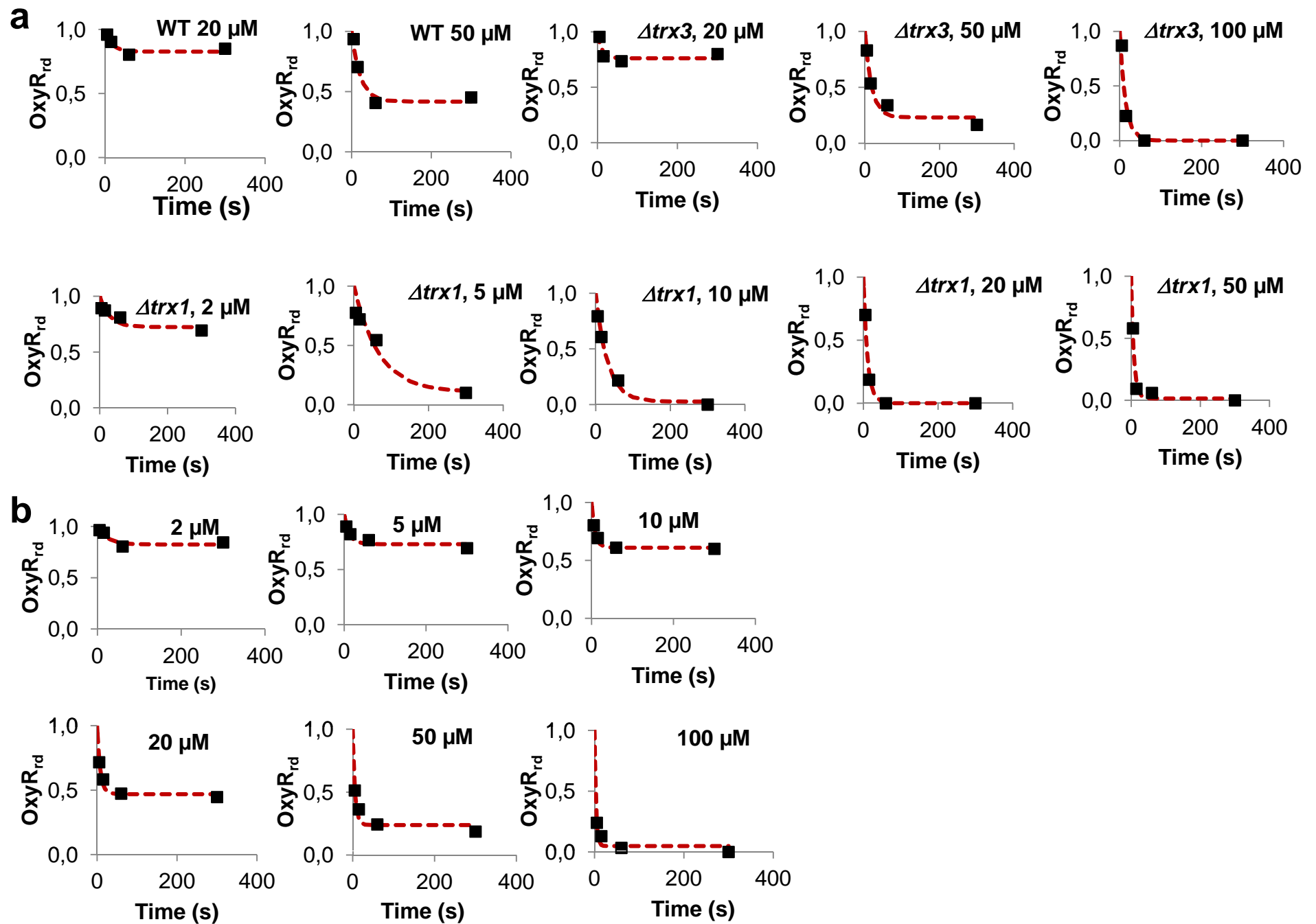

Supplement: Supplementary file 3 — Figure S3. Determination of gradients between extracellular and intracellular H2O2 concentrations. Gradients are obtained from the non-linear fitting of Eq. 1 (dashed lines) to OxyR oxidation measured experimentally (filled squares) after adding the indicated concentration of H2O2. Experiments were done under aerobic conditions with wild-type, Δtrx1, or Δtrx3 strains (a) or under anaerobic conditions in the Δtpx1 strain (b). (PDF 150 kb) [file 12915_2018_523_MOESM3_ESM.pdf]

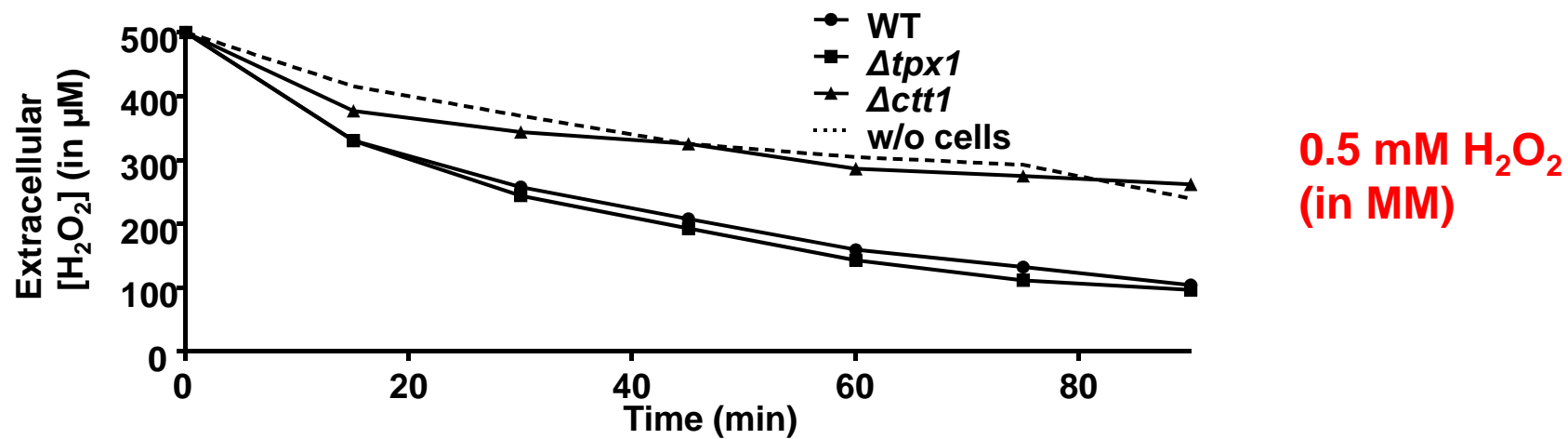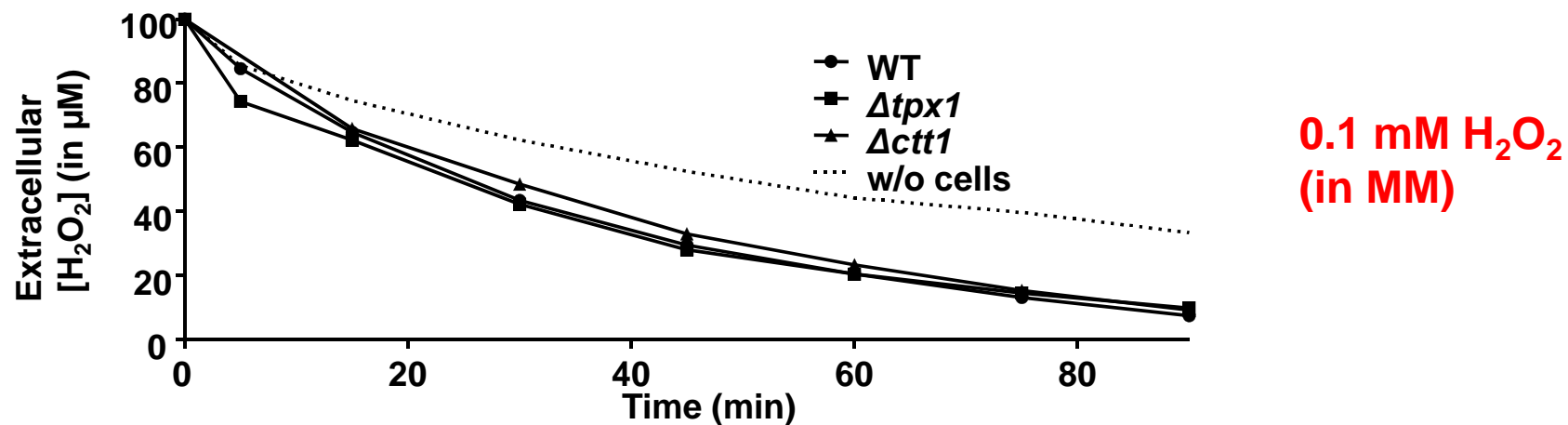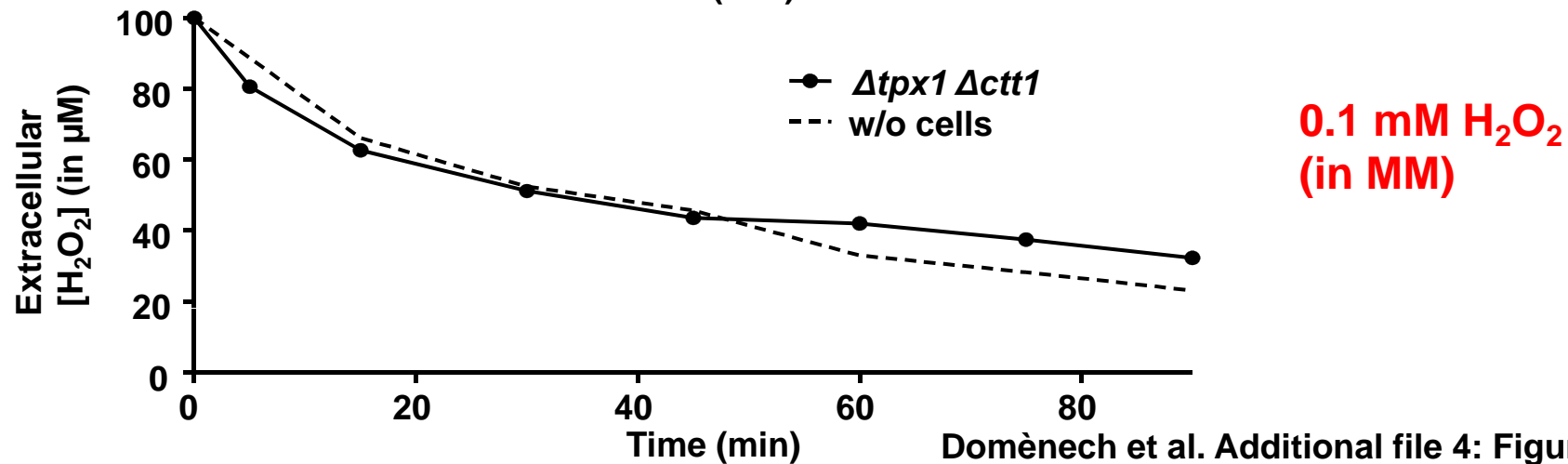

Supplement: Supplementary file 4 — Figure S4. Both Tpx1 and Ctt1 participate in peroxide scavenging at sub-toxic doses of H2O2, while only catalase acts as a scavenger of the toxic ones. The concentration of the remaining extracellular peroxides of MM cultures of strains 972 (WT), SG4 (Δtpx1), EP198 (Δctt1), and SG267 (Δtpx1 Δctt1), treated with 0.1 or 0.5 mM H2O2, was determined at the times indicated with a colorimetric reaction (see Methods). (PDF 111 kb) [file 12915_2018_523_MOESM4_ESM.pdf]

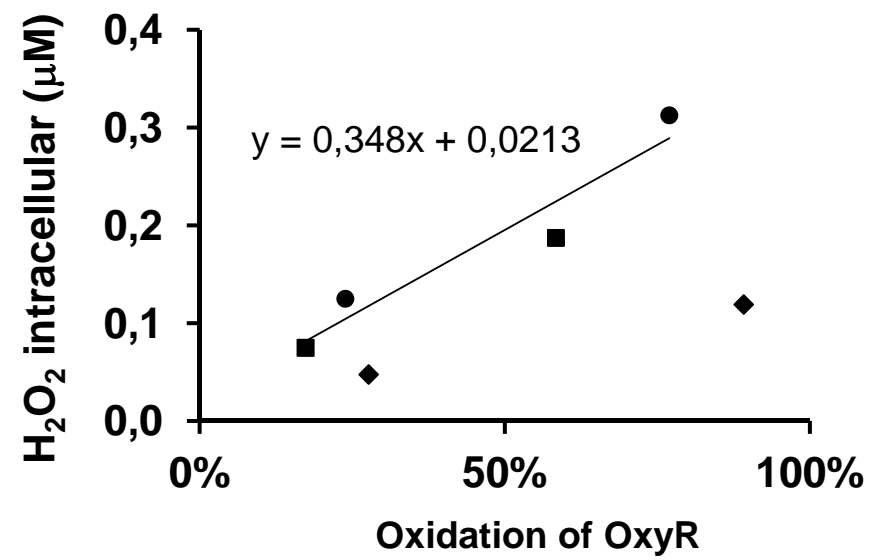

Domènech et al. Additional file 5: Figure S5

Supplement: Supplementary file 5 — Figure S5. OxyR as a sensor of intracellular H2O2 concentrations. The relation between intracellular H2O2 concentration and OxyR oxidation levels observed at steady state after addition of H2O2 to the WT (filled squares), Δtrx1 (filled diamonds), or Δtrx3 (filled circles) strains is plotted according to the data shown in Table 1 in the main text. (PDF 17 kb) [file 12915_2018_523_MOESM5_ESM.pdf]

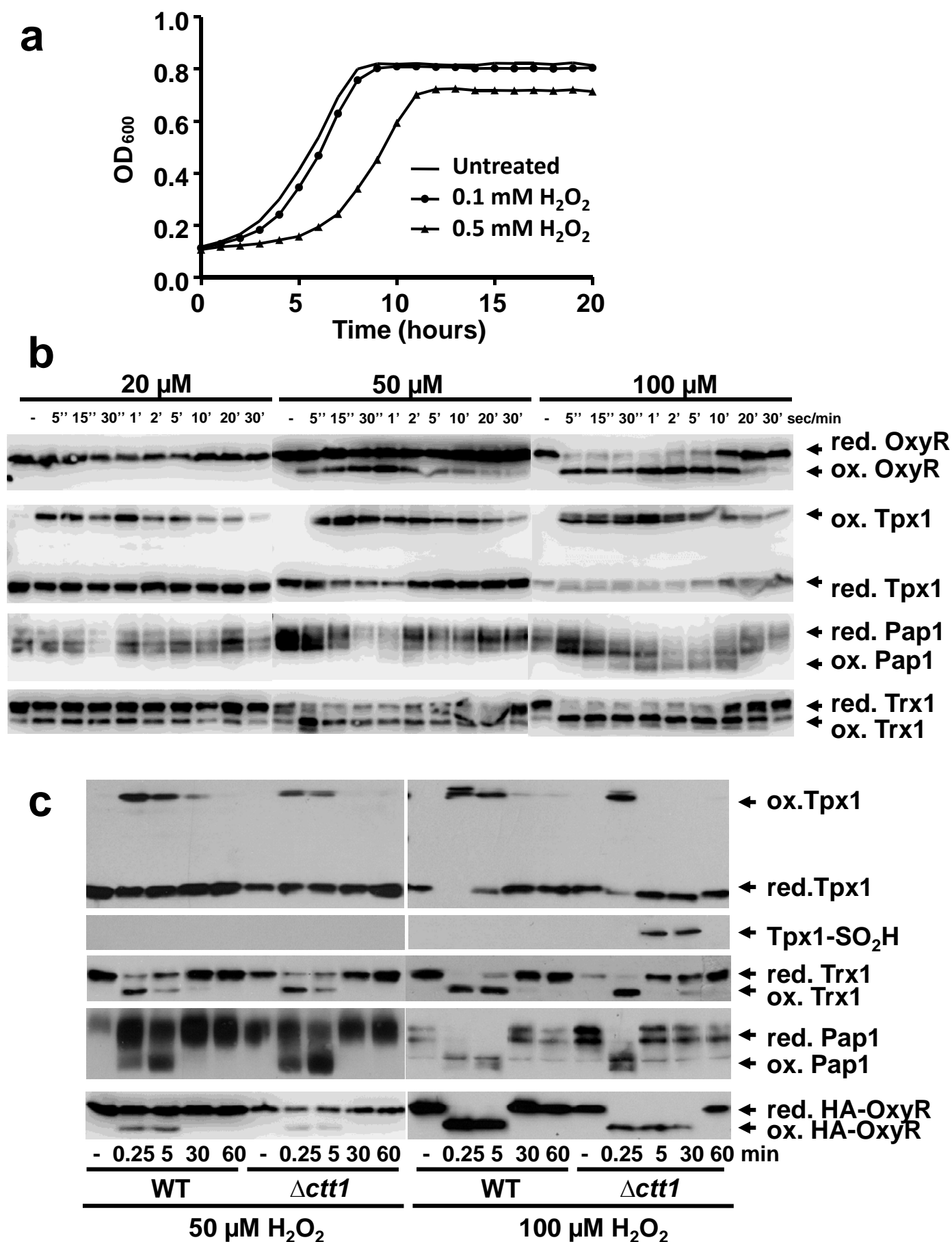

Domènech et al. Additional file 6: Figure S6

Supplement: Supplementary file 6 — Figure S6. At non-toxic concentrations of extracellular peroxides, both systems, Pap1-Tpx1 and OxyR, behave similarly. a Growth curves of strain AD29 (WT + HA-OxyR), treated or not with 0.1 mM or 0.5 mM H2O2, were recorded for 20 h. b Cultures of strain AD29 (WT + HA-OxyR) were treated or not with the indicated concentrations of H2O2 for the times indicated, and protein extracts were obtained and processed as described in Fig. 2a, using antibodies against HA (red. HA-OxyR and ox. HA-OxyR), Tpx1 (ox. Tpx1 dimer and red. Tpx1 monomer), Pap1 (red. Pap1 and ox. Pap1), or Trx1 (red. Trx1 and ox. Trx1). c Catalase acts as a scavenger at 100 μM, but not at 50 μM H2O2. Cultures of strains AD29 (WT) and AD163 (Δctt1), carrying an integrative sty1 promoter-driven HA-oxyR gene, were treated or not with 50 or 100 μM H2O2 for the times indicated, and protein extracts were obtained and processed as described in Fig. 2a, using antibodies against Tpx1 (ox. Tpx1 dimer and red. Tpx1 monomer), sulfinylated Prx (Tpx1-SO2H), Trx1 (red. Trx1 and ox. Trx1), Pap1 (red. Pap1 and ox. Pap1), or HA (red. HA-OxyR and ox. HA-OxyR). (PDF 281 kb) [file 12915_2018_523_MOESM6_ESM.pdf]

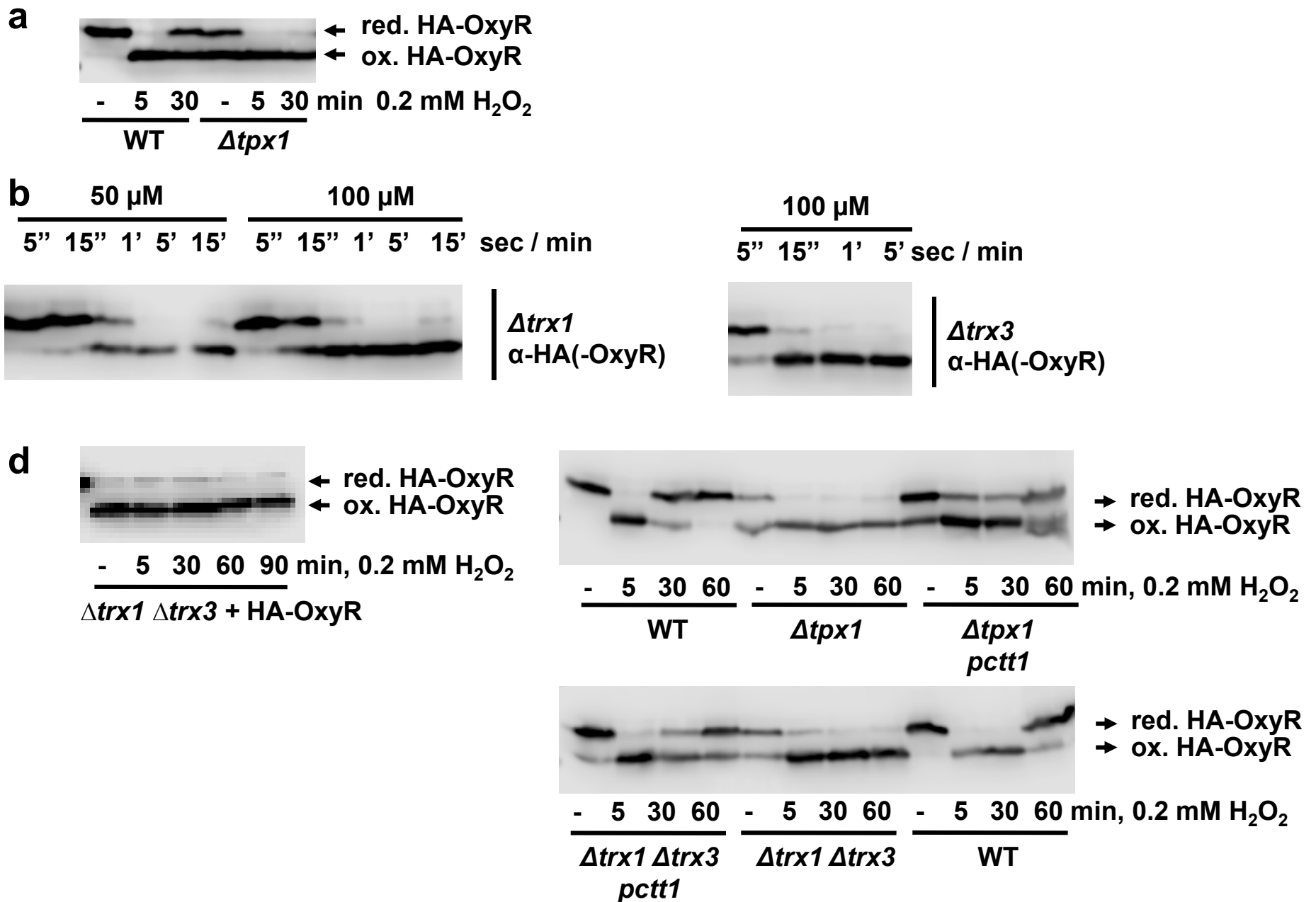

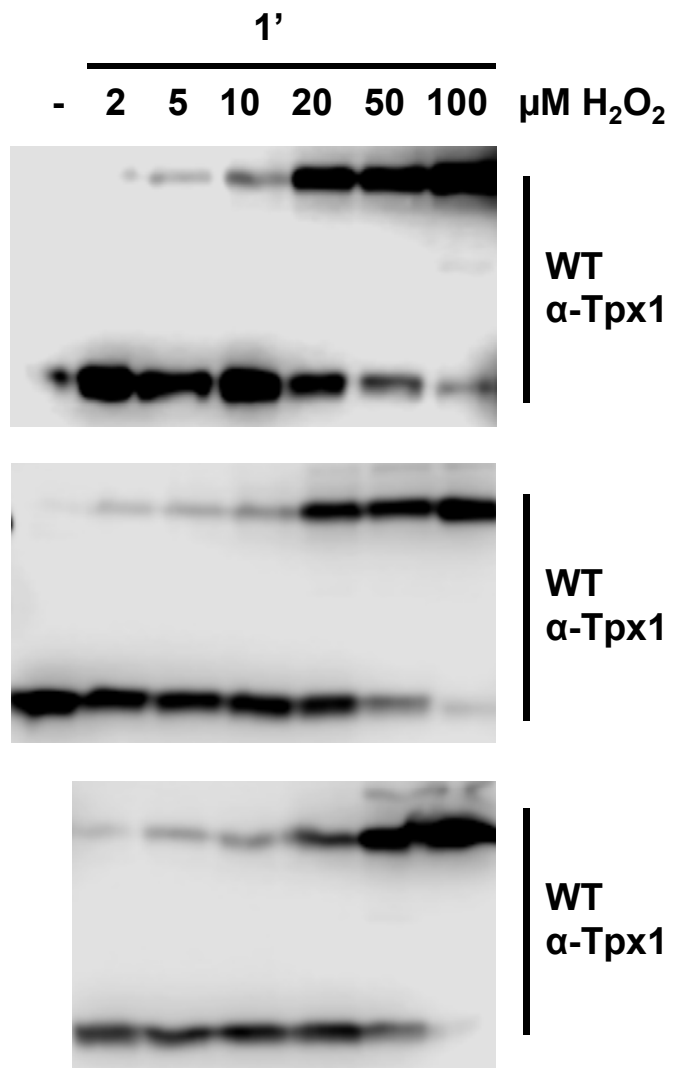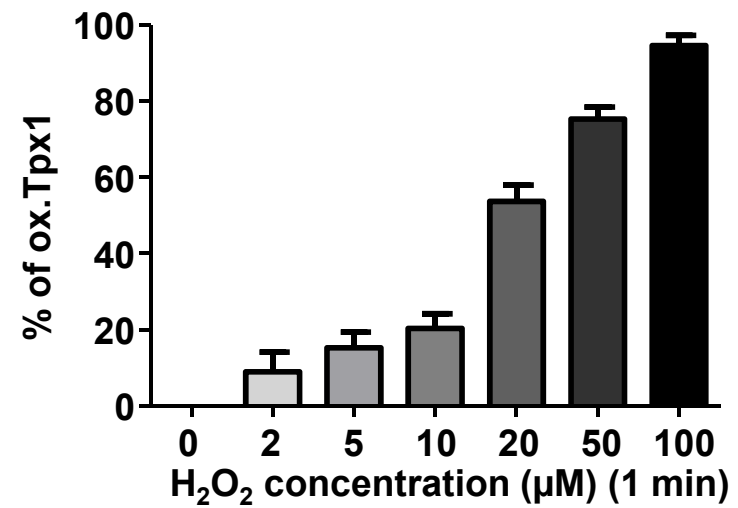

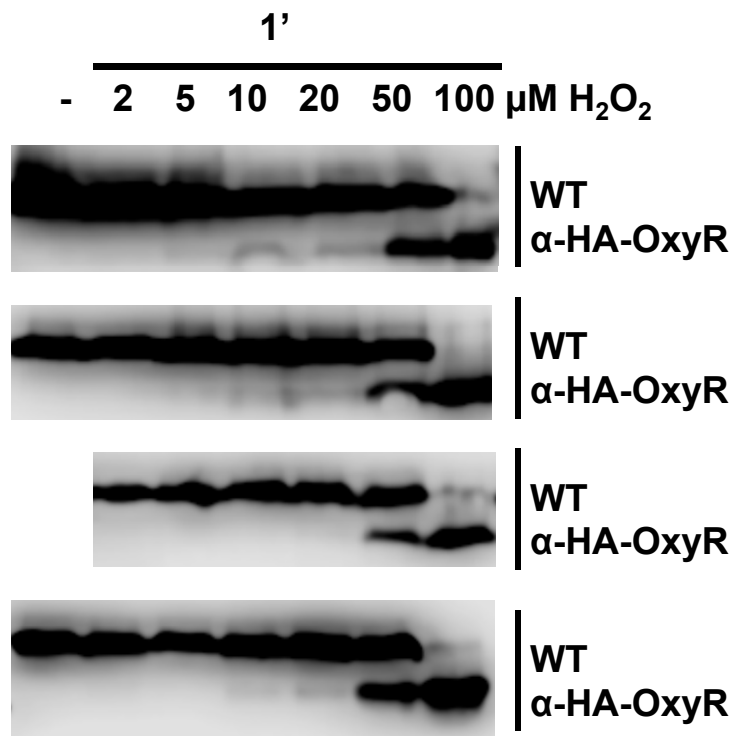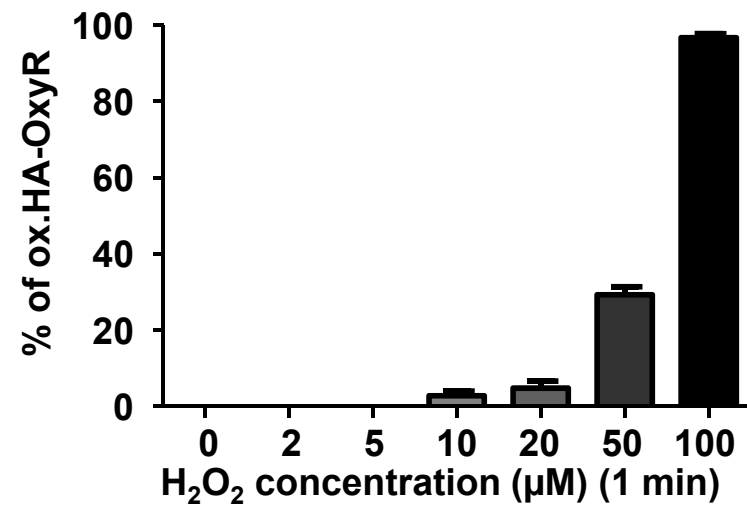

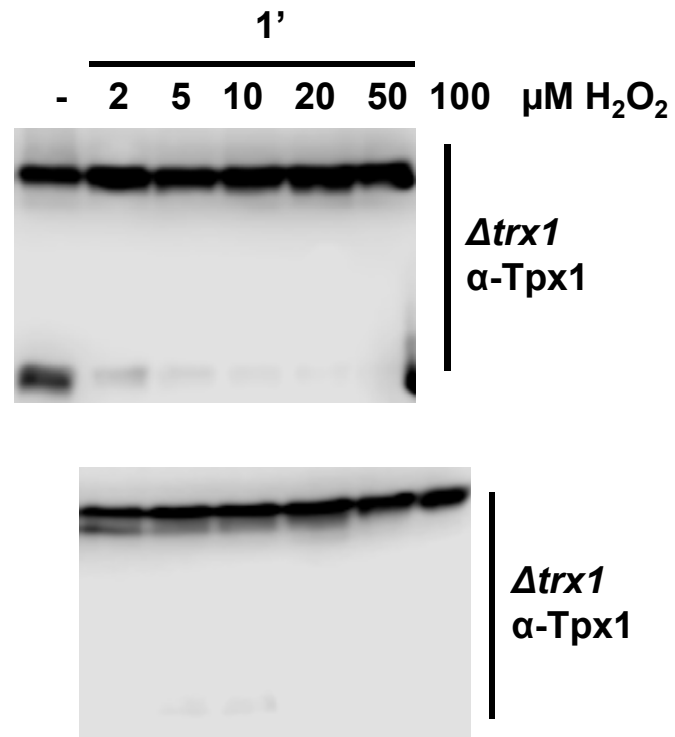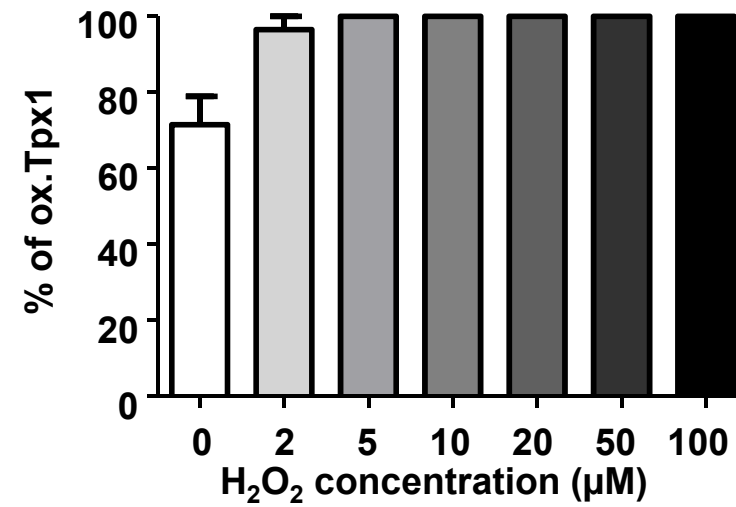

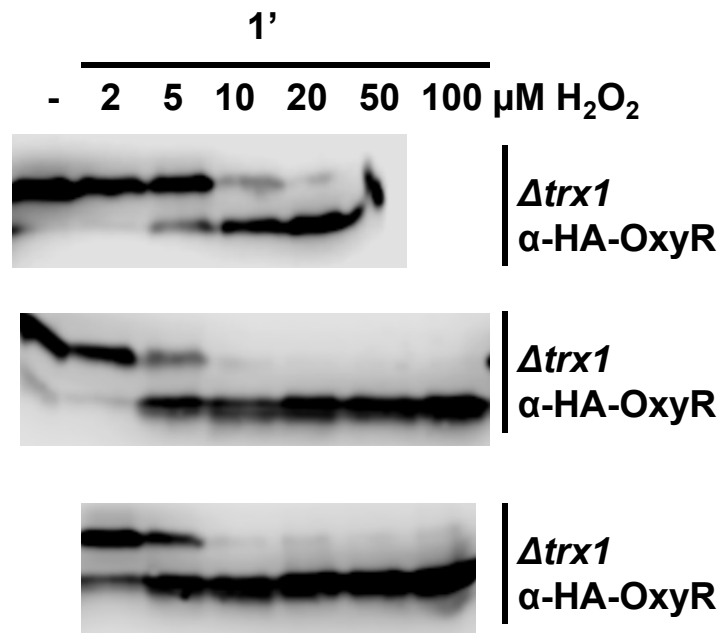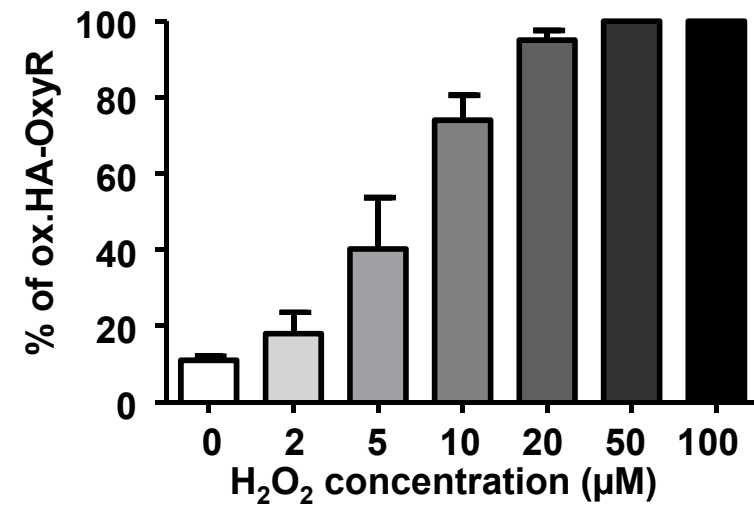

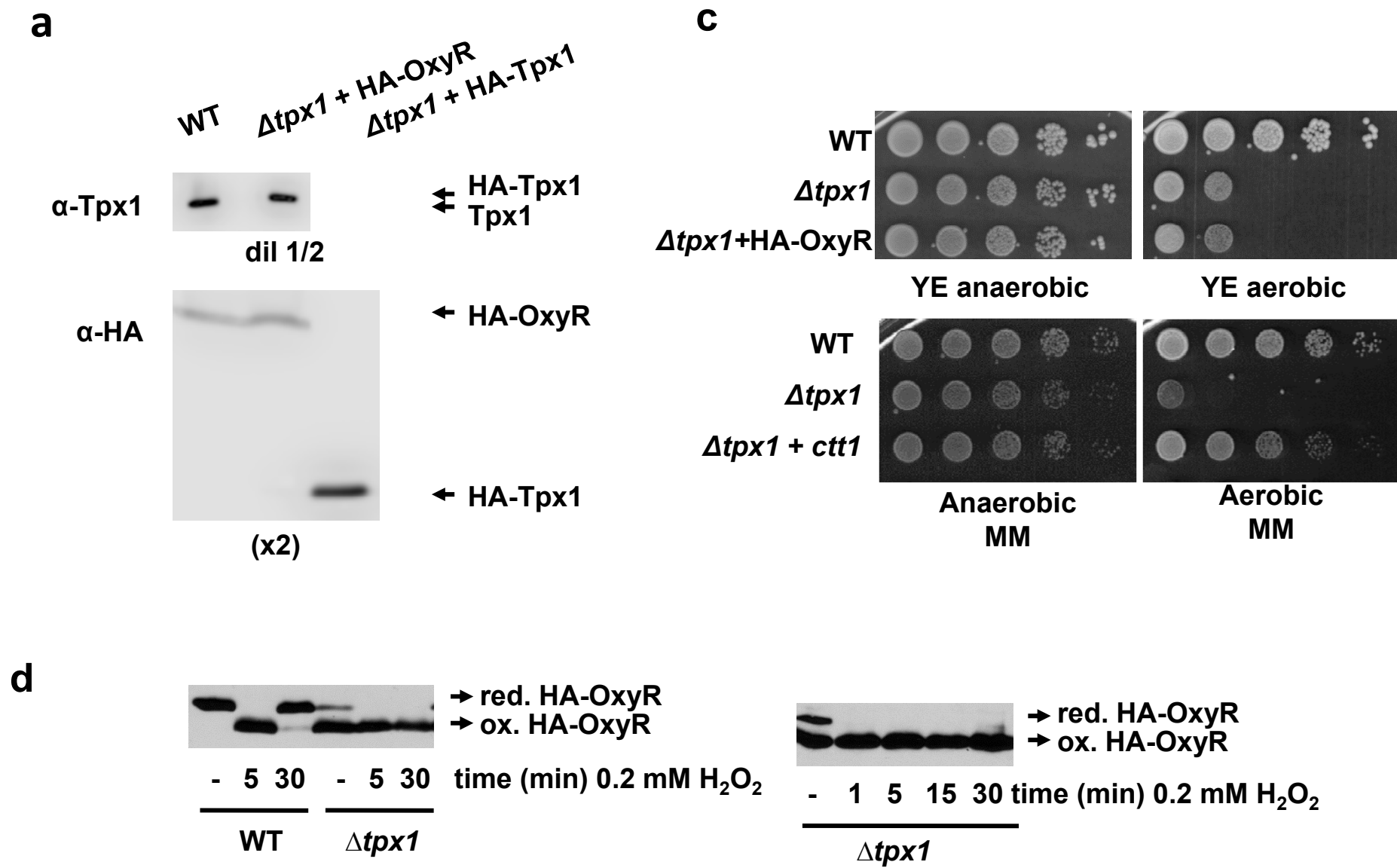

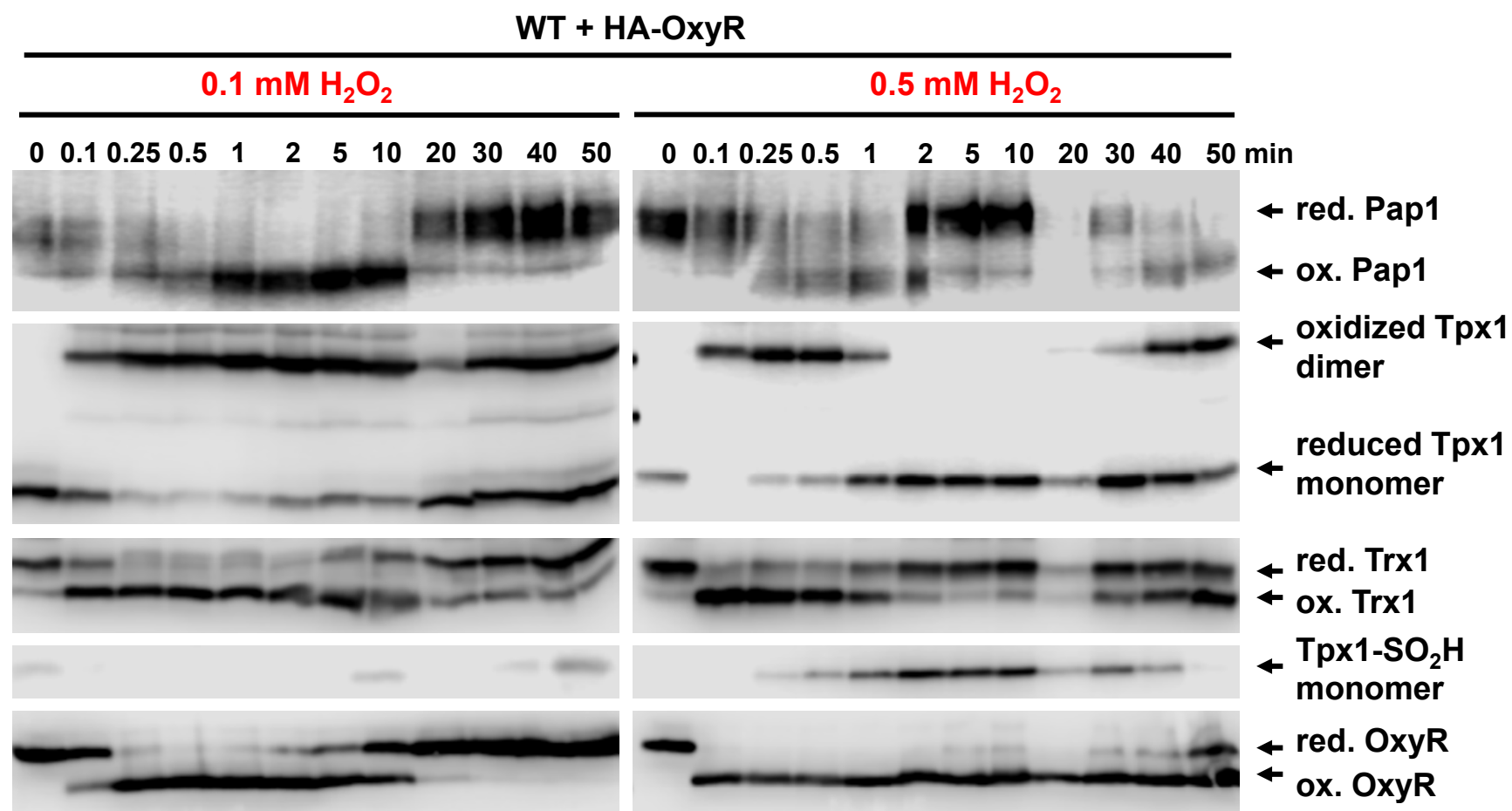

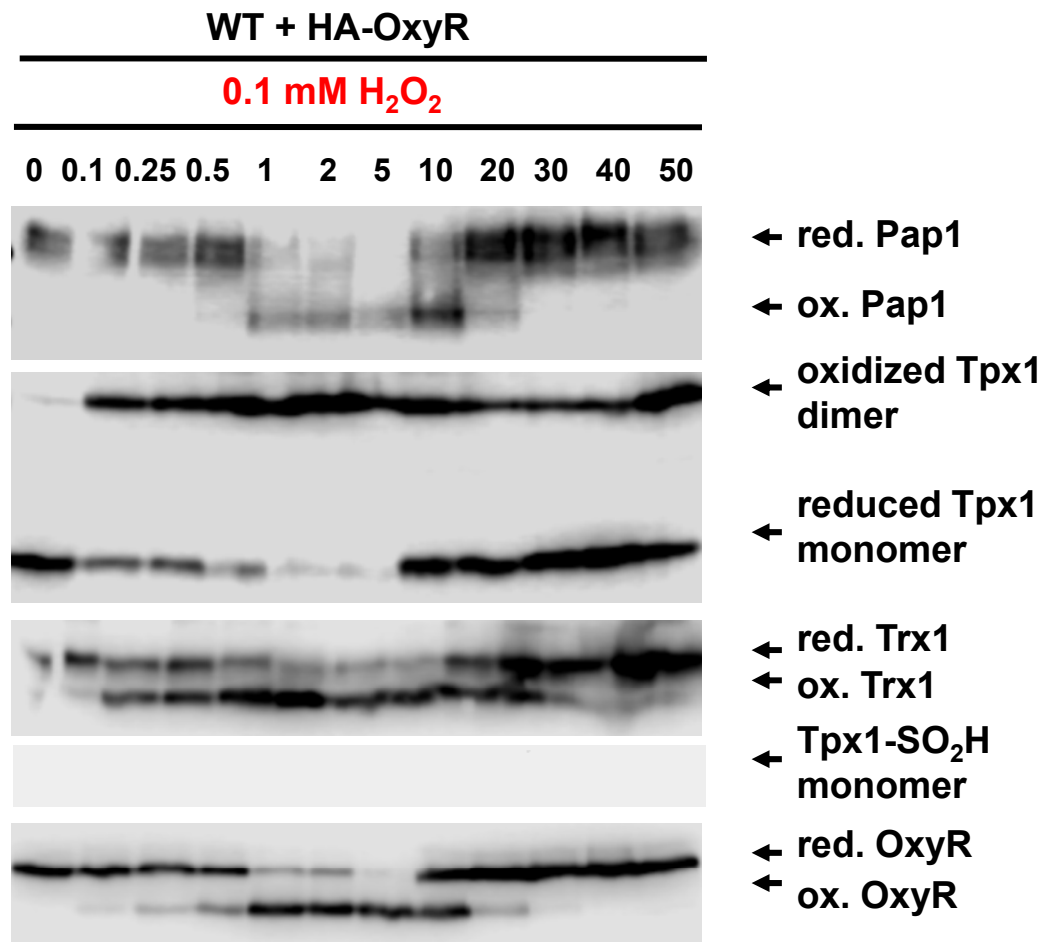

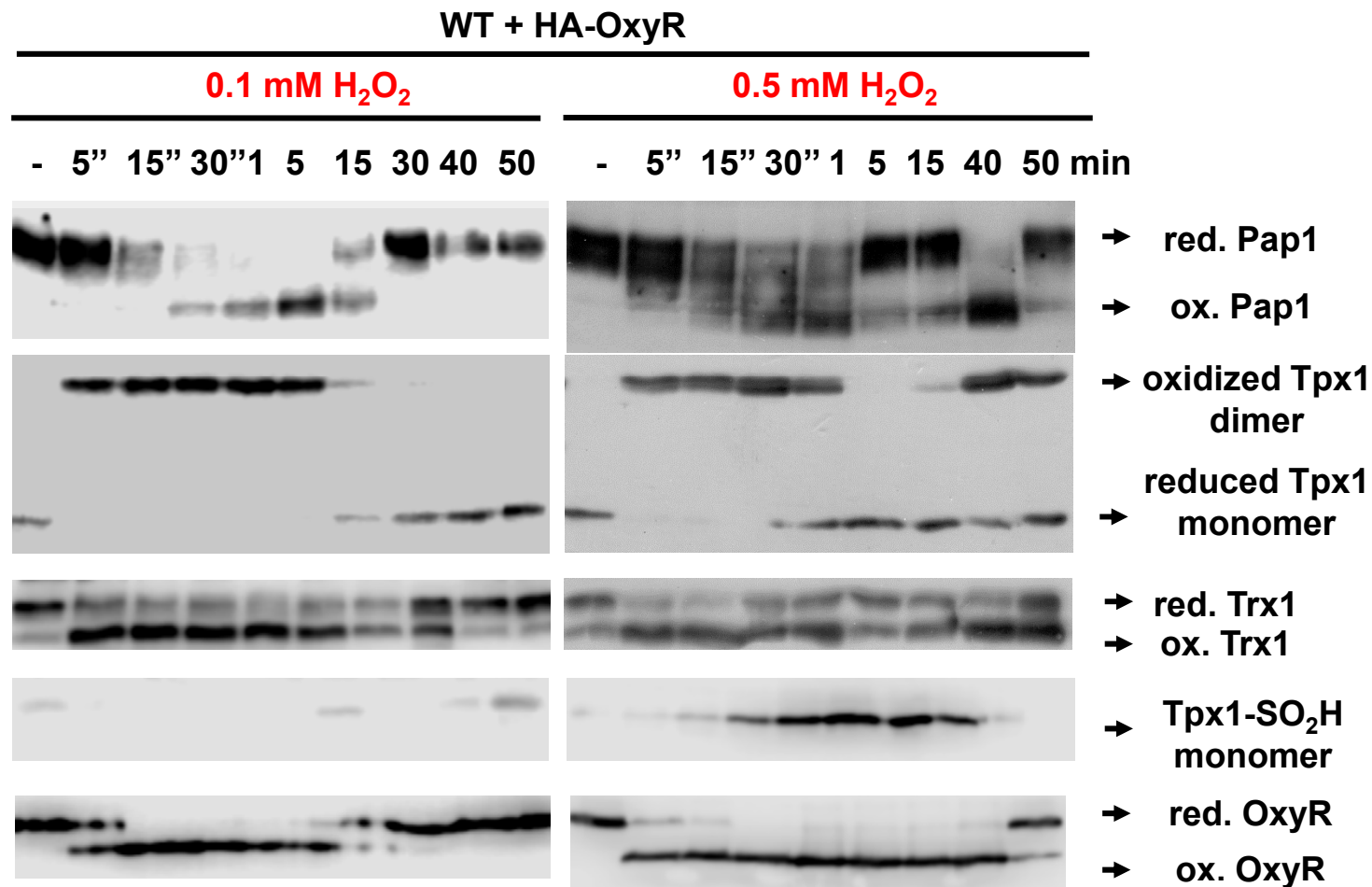

Supplement: Supplementary file 8 — Figure S7. Biological replicates of main figures. (PDF 1383 kb) [file 12915_2018_523_MOESM8_ESM.pdf]
