# Supplementary material for: Using in vivo oxidation status of one- and two-component redox relays to determine H2O2 levels linked to signaling and toxicity
Source: BMC Biol. 2018 Jun 1;16:61. doi: 10.1186/s12915-018-0523-6 (PMC5984441; doi:10.1186/s12915-018-0523-6)
Supplement: Supplementary file 7 — Table S1. Strains used in this study. (PDF 10 kb) [file 12915_2018_523_MOESM7_ESM.pdf]

Table S1. Strains used in this study

Strain	Genotype	Origin
972	h-	[1]
SG5	*h^+ tpx1::natMX6*	[2]
SG4	*h^+ tpx1::natMX6*	[3]
EP198	*h^+ ctt1::natMX6*	[4]
SG267	*h^+ ctt1::ura4 tpx1::kanMX6 ura4-D18*	[4]
AD7	*h^+ tpx1::natMX6 sty1'::ctt1::leu1*	[4]
AD29	*h^- sty1'::HA-oxyR::leu1*	This work
AD36	*h^- tpx1::natMX6 sty1'::HA-oxyR::leu1*	This work
AD47	*h^- trx3:: hphMX6 sty1'::HA-oxyR::leu1 ura4-D18*	This work
AD58	*h^- tpx1::natMX6 gpx1::kanMX6 pmp20::bleMX6 BCP::hphMX6 sty1'::HA-oxyR::leu1*	This work
AD59	*h^- grx1::kanMX6 sty1'::HA-oxyR::leu1*	This work
AD61	*h^+ trx1::kanMX6 sty1'::HA-oxyR::leu1 ura4-D18*	This work
AD62	*h^- trx1::kanMX6 trx3::natMX6 sty1'::HA-oxyR::leu1*	This work
AD65	*h^+ trr1::natMX6 sty1'::HA-oxyR::leu1*	This work
AD66	*h^+ tpx1::natMX6 trr1:: ura4 sty1'::HA-oxyR::leu1*	This work
AD94	*h^+ tpx1::kanMX6 sty1'::HA-oxyR::leu1 ura4-D18 ade6-M210*	This work
AD98	*h^+ trx1::natMX6 trx3::kanMX6 sty1'::HA-oxyR::leu1 ura4-D18*	This work
AD106	*h^- pgr1::natMX6 sty1'::HA-oxyR::leu1*	This work
AD133	*h^+ pap1::ura4 tpx1::natMX6 trr1::kanMX6 sty1'::HA-oxyR::leu1 ade6*	This work
AD163	*h^- ctt1::ura4 sty1'::HA-oxyR::leu1*	This work

SUPPLEMENTARY REFERENCES

1. Leupold U: Genetical methods for *Schizosaccharomyces pombe*. Methods Cell Physiol 1970;4:169-177.
2. Calvo IA, Boronat S, Domenech A, Garcia-Santamarina S, Ayte J, Hidalgo E: Dissection of a redox relay: H2O2-dependent activation of the transcription factor Pap1 through the peroxidatic Tpx1-thioredoxin cycle. Cell Rep 2013;5(5):1413-1424.
3. Boronat S, Domenech A, Carmona M, Garcia-Santamarina S, Bano MC, Ayte J, Hidalgo E: Lack of a peroxiredoxin suppresses the lethality of cells devoid of electron donors by channelling electrons to oxidized ribonucleotide reductase. PLoS Genet 2017;13(6):e1006858.
4. Paulo E, Garcia-Santamarina S, Calvo IA, Carmona M, Boronat S, Domenech A, Ayte J, Hidalgo E: A genetic approach to study H2O2 scavenging in fission yeast--distinct roles of peroxiredoxin and catalase. Mol Microbiol 2014;92(2):246-257.
